# Circular RNA hsa_circ_0043278 inhibits breast cancer progression via the miR-455-3p/EI24 signalling pathway

**DOI:** 10.1186/s12885-021-08989-w

**Published:** 2021-11-20

**Authors:** Yue Shi, Chong Liu

**Affiliations:** 1 Department of Geriatric Surgery, The First Affiliated Hospital of China Medical University, Shenyang, 110001 China; 2 Department of Breast Surgery, The First Affiliated Hospital of China Medical University, Shenyang, 110001 China

**Keywords:** Breast cancer, Circular RNA, hsa_circ_0043278

## Abstract

**Background:**

Breast cancer (BC) is one of the major malignancies worldwide. Circular ribonucleic acids (circRNAs) are a class of conserved ribonucleic acid (RNA) molecules that play important roles in various diseases. Recently, circRNAs have been suggested to have diagnostic value and may function as potential diagnostic biomarkers for BC. Previously, hsa_circ_0043278 was found to be downregulated in human BC. However, its role in human BC has not yet been identified.

**Methods:**

The levels of hsa_circ_0043278 in BC cell lines were verified by quantitative reverse transcription–polymerase chain reaction (qRT–PCR). The overexpression vector and short hairpin RNA (shRNA) of hsa_circ_0043278 were transfected into MDA-MB-231 and MCF-7 cells, respectively. The effects of hsa_circ_0043278 on tumour cell growth, migration and invasion were measured by 3-(4,5-Dimethylthiazol-2-yl)-2,5-diphenyl-tetrazolium bromide (MTT), colony formation, wound healing and Transwell assays *in vitro*. A xenograft experiment was conducted to validate the inhibitory effect of hsa_circ_0043278 on tumour growth. The interaction between hsa_circ_0043278 and miR-455-3p was confirmed by a dual-luciferase reporter assay. Mimics and inhibitors of miR-455-3p were designed to confirm the influence of hsa_circ_0043278 on the hsa_circ_0043278/miR-455-3p/etoposide-induced gene 24 (EI24) axis.

**Results:**

Hsa_circ_0043278 was downregulated in BC cell lines. Furthermore, overexpression of hsa_circ_0043278 notably decreased BC cell viability and inhibited BC cell migration and invasion *in vitro* and suppressed tumour growth *in vivo*. Downregulation of hsa_circ_0043278 led to the opposite results. Hsa_circ_0043278 expression was negatively correlated with that of miR-455-3p. In addition, mechanistic investigation proved that hsa_circ_0043278 directly bound to miR-455-3p and regulated EI24 and NF-κB expression in BC cells.

**Conclusion:**

Hsa_circ_0043278 acts as a tumour suppressor gene in BC through the hsa_circ_0043278/miR-455-3p/EI24 axis and may be regarded as a new prognostic predictor or potential therapeutic target in BC.

**Supplementary Information:**

The online version contains supplementary material available at 10.1186/s12885-021-08989-w.

## Background

Breast cancer (BC) is the second most common cancer worldwide and is a major cause of female death [1, 2]. Some classical biomarkers, such as CEA and CA 15-3, detected in serum or nipple discharge, are associated with the development of BC [3, 4]. But their tumour specificity is unsatisfactory. Furthermore, studies show that several genetic variants are potentially associated with BC risk, which provides insights into new potential biomarkers for BC [5, 6]. However, biomarkers for the diagnosis and prognosis of human BC remain limited [7]. Hence, it is still important to understand the underlying molecular processes of BC to explore novel biomarkers for BC progression and therapeutic targets.

Circular ribonucleic acids (circRNAs) are a class of stable, conserved ribonucleic acid (RNA) molecules [8, 9]. Recent studies have indicated that circRNAs encode small peptides [10, 11]. Most studies have proven that circRNAs act as sponges of microRNAs (miRNAs) to further regulate the development of multiple human diseases [8, 12–16]. In human BC, many circRNAs have been discovered and proven to be related to the development of BC [17]. Notably, a meta-analysis indicated that circRNAs have diagnostic value and may be potential diagnostic biomarkers for BC [18]. However, research on the mechanisms of circRNAs in BC progression remains limited. In our previous study, a novel circRNA, hsa_circ_0043278, was identified and found to be downregulated in BC tissues [19]. In addition, we predicted five putative target miRNAs. However, the underlying mechanisms of hsa_circ_0043278 remain to be elucidated. Thus, these findings prompted our interest in revealing the association between hsa_circ_0043278 and BC development.

In the present study, the low expression of hsa_circ_0043278 in BC cell lines was examined. Moreover, its target miRNA, miR-455-3p, has been considered an oncogene in BC owing to its inhibitory effect on etoposide-induced gene 24 (EI24) expression [20]. EI24 has also been reported to have a role in suppressing tumour progression by inhibiting NF-κB activity [21]. Hence, based on the aforementioned information, we hypothesized that hsa_circ_0043278 might influence BC development by targeting miR-455-3p and regulating EI24. Collectively, our results showed that hsa_circ_0043278 might act as a tumour suppressor gene in BC progression. Exploration of its molecular mechanism could be valuable for BC diagnosis and therapy.

## Materials and methods

### Tissue specimens

A total of 50 pairs of BC tissues and matched normal tissues were collected from BC patients at The First Affiliated Hospital of China Medical University (from January 2005 to December 2012). All patients were diagnosed with primary BC, female, between 27 and 81 years old, and did not receive chemotherapy or radiotherapy before surgery. Tissue specimens were obtained after surgical resection and stored at -80 °C in an ultralow-temperature freezer (Haier, China). The matched normal tissues were collected from regions more than 5 cm outside the edge of BC tissues. The study was approved by the Ethics Committee of The First Affiliated Hospital of China Medical University. All procedures performed in this study involving specimen collection and experiments were in accordance with the Declaration of Helsinki. Written informed consent was obtained from all patients.

### Cell culture

Human BC cell lines (MDA-MB-231 and MCF-7) and a normal mammary epithelial cell line (MCF-10A) were purchased from the Cell Bank of the Chinese Academy of Sciences. The MDA-MB-468, BT-549, SK-BR-3, T47D and HEK 293T cell lines were maintained in our laboratory. MDA-MB-231 cells were cultured in Dulbecco’s modified Eagle’s medium (DMEM; Gibco, Carlsbad, CA, USA) containing 10% foetal bovine serum (FBS; HyClone, Logan, UT, USA). MCF-7 cells were cultured in minimum essential medium (MEM; Gibco) containing 10% FBS and 0.01 mg/ml insulin from bovine pancreas (Aladdin, Shanghai, China). Michigan Cancer Foundation-10A (MCF-10A) cells were cultured in mammary epithelial basal medium (MEBM; iCell Bioscience, Shanghai, China). The other cell lines were cultured appropriately. All these cell lines were maintained at 37 °C in a humidified incubator with 5% carbon dioxide (CO_2_).

### qRT–PCR

Total RNA from tissues or cells was extracted with a simple Total RNA Kit (BioTeke, Beijing, China) following the manufacturer’s instructions. The purity and concentration of the RNA were determined using a NanoDrop 2000 spectrophotometer (Thermo Scientific, Waltham, MA, USA). Complementary deoxyribonucleic acid (cDNA) was reverse transcribed from the total RNA using Moloney Murine Leukaemia Virus (M-MLV) Reverse Transcriptase (BioTeke). Quantitative reverse transcription–polymerase chain reaction (qRT–PCR) was performed in an Exicycler^TM^ 96 RT-PCR instrument (BIONEER, Daejeon, Korea) using the SYBR Green method (Takara Bio, Dalian, China). β-Actin was used as the control for the normalization of circRNA and messenger RNA (mRNA) levels, while U6 was used for normalization of miRNA levels. Table S1 shows the primers for gene amplification, which were designed with Primer Premier 5 (PREMIER Biosoft, USA) and synthesized by Sangon Biotech (Shanghai, China). The relative expression of genes was analysed using the 2^-△CT^ method. All the experiments were repeated three times.

### Vector construction and cell transfection

To overexpress hsa_circ_0043278, the mature sequence of hsa_circ_0043278 (chr17, 35797838–35800763) was synthesized and then cloned into the pCD5-ciR vector (GenScript, Nanjing, China) and was named “ov-circ”. A mock vector without the hsa_circ_0043278 sequence (ov-NC) served as the negative control. To knock down hsa_circ_0043278, two small interfering RNAs (siRNAs) targeting the back-splice junction site of hsa_circ_0043278 and a negative control siRNA (si-NC) were synthesized (GenScript). Consequently, qRT–PCR proved that siRNA-2 was the most effective siRNA, and it was used to construct the siRNA plasmid (Fig. S1). The shRNA against hsa_circ_0043278 and the negative control shRNA were synthesized and cloned into the pRNAH1.1 vector, and the resulting vectors were named “sh-circ” and “sh-NC”, respectively. The sequences of the vectors and siRNAs were designed with the online NCBI database (https://www.ncbi.nlm.nih.gov/). The miR-455-3p mimics and inhibitors were purchased from GenePharma (Shanghai, China). Vectors, mimics, and inhibitors were transiently transfected into BC cells with Lipofectamine 2000 (Invitrogen, Carlsbad, CA, USA) following the manufacturer’s protocols. The sequences of the siRNAs and shRNAs are shown in Table S2. The miR-455-3p inhibitor and negative control sequences are listed in Table S3.

### Western blotting

Proteins from tissues and cells were extracted using radioimmunoprecipitation assay (RIPA) lysis buffer (Beyotime, Haimen, China) supplemented with 1 mM phenylmethylsulfonyl fluoride (PMSF; Beyotime). A total of 40 μg (in 20 μl) of protein from each sample was separated by sodium dodecyl sulfate–polyacrylamide gel electrophoresis (SDS–PAGE) and was then transferred onto polyvinylidene difluoride (PVDF) membranes (Millipore, Bedford, MA, USA). Next, the membranes were incubated with 5% milk at room temperature for 1 h. Then, they were incubated with the appropriate primary antibody overnight at 4 °C and then with a horseradish peroxidase-conjugated secondary antibody (1:5,000; Wanleibio, Shenyang, China). The specific primary antibodies included anti-EI24 (1:1000, Proteintech, Wuhan, China) and anti-NF-κB (P65) (1:500, Wanleibio) antibodies. β-Actin and histone H3 (1:1000, Wanleibio) served as the internal controls. Immune complexes were finally visualized with an enhanced chemiluminescence system (Beyotime).

### 3-(4,5-Dimethylthiazol-2-yl)-2,5-diphenyl-tetrazolium bromide (MTT) assay

Cells were seeded in 96-well plates at a density of 3×10^3^ cells per well. MTT solution (5 mg/mL; Sigma-Aldrich, St. Louis, Missouri) was added to each well at the indicated time points (0, 24, 48, and 72 h), and the plates were incubated at 37 °C in 5% CO_2_ for 4 h. Next, the medium was removed, and 200 μL of dimethyl sulfoxide (DMSO) was added to dissolve the formazan crystals. The optical density at 570 nm was recorded using an automatic microplate reader (BioTek, Vermont, USA).

### Colony formation assay

Briefly, cells in each group were seeded in 35-mm dishes at 500 cells/dish and incubated at 37 °C in 5% CO_2_ for 2 weeks. The cells were fixed and stained with Giemsa solution (KeyGEN BioTECH, Jiangsu, China). Colonies consisting of at least 50 cells were counted and photographed under a microscope.

### Wound healing assay

Cells were seeded (6×10^5^ cells/well) and grown in 6-well plates for 48 h post transfection. Next, the cells were cultured in serum-free culture medium and treated with 1 μg/ml mitomycin C (Sigma-Aldrich) for 1 h. Wounds were created in the middle of the wells using 200-μL pipette tips. The cells were then washed and cultured in serum-free culture medium, and wound closure was monitored by imaging with a phase contrast microscope (Motic, Xiamen, China). After culture at 37 °C in 5% CO_2_ for 48 h, the widths of the wounds were measured again, and the migration rates were calculated in each group.

### Migration and invasion assays

For the migration assays, 2×10^4^ cells were seeded in the upper chambers of a Transwell plate (Corning Incorporated, Corning, NY, USA) in 200 μL of serum-free medium. An 800-μL volume of medium containing 20% FBS was added to the lower chambers. The pore size of the Transwell membrane was 8.0 μm. After incubation at 37 °C in 5% CO_2_ for 24 h, the cells that migrated to the bottom of the filter were fixed with 4% paraformaldehyde and stained with 0.5% crystal violet (Amresco, USA) and were then photographed and counted under an inverted phase contrast microscope (Motic).

For the invasion assays, 2×10^4^ cells in 200 μL of serum-free medium were seeded in the upper chambers of a Transwell plate with a Matrigel-coated membrane (BD Biosciences, NJ, USA). An 800-μL volume of medium containing 20% FBS was added to the lower chambers. The pore size of the Transwell membrane was 8.0 μm. After incubation at 37 °C in 5% CO_2_ for 24 h, the cells in the lower compartment were fixed with 4% paraformaldehyde and stained with 0.5% crystal violet (Amresco) and were then photographed and counted under an inverted phase contrast microscope (Motic).

### Xenograft experiment

A total of 18 two-month-old female BALB/c (nu/nu) mice were purchased from Wanleibio (Shenyang, China). The animal care and experimental procedures were approved by the Experimental Animal Ethics Committee of The First Affiliated Hospital of China Medical University. All animal procedures were performed in accordance with the Guide for the Care and Use of Laboratory Animals published by the National Institutes of Health (NIH, Bethesda, Maryland, USA). We made great attempts to reduce the pain experienced by the animals. Mice were adaptively fed for 1 week under specific pathogen-free conditions. To establish xenograft tumours, 0.2 mL (5 × 10^7^ cells/ml) of MDA-MB-231 cells stably transfected with the hsa_circ_0043278 overexpression vector or MCF-7 cells stably transfected with hsa_circ_0043278 shRNA was subcutaneously injected into the right axillae of the mice, and mice injected with mock vector or shRNA served as negative controls (*n*=3 mice in each group; 18 mice total). The tumour sizes were measured once a week with a calliper. The tumour volumes were calculated using the following formula: 1/2 (length × width^2^). In brief, mice were sacrificed in the 4th week, and the tumours were then excised, photographed and weighed.

### Immunohistochemistry (IHC)

The expression of NF-κB (P65) in the paraffin-embedded xenograft tissue was detected by immunohistochemistry. Tissues were incubated with the anti-NF-κB (P65) (1:200; Wanleibio) primary antibody at 4 °C overnight and then with the secondary antibody at 37 °C for 60 min and an HRP-labelled streptavidin solution for 10 min. Next, tissues were stained by diaminobenzidine (DAB) (Solarbio, China) and observed under a microscope. The mean density of P65 in the tissues was calculated using Image-Pro software.

### Dual-luciferase reporter assay

The sequences of wild-type hsa_circ_0043278 or a mutant without miR-455-3p binding sites were synthesized and subcloned into the luciferase reporter vector pmirGLO (GenScript); the resulting vectors were named “circ-WT” and “circ-Mut”, respectively. HEK 293T cells were cotransfected with the vectors and miR-455-3p mimics or negative controls. Relative luciferase activity was measured with a Dual-Luciferase Assay Kit (KeyGEN BioTECH) in accordance with the manufacturer’s protocols after 48 h of incubation. Luciferase activity was measured using a Tecan Infinite M200 Pro luminometer (Tecan, Männedorf, Switzerland).

### Statistical analysis

Statistical analysis was performed using GraphPad Prism 7.0 software (GraphPad Software, La Jolla, CA, USA). The data for two groups were compared using a *t* test with the Wilcoxon signed-rank test. Comparisons of the means among multiple groups were performed by one-way analysis of variance (ANOVA) with the Kruskal–Wallis test. Pearson correlation coefficients were calculated for correlation analyses. Continuous data are presented as the mean ± standard deviation values (SD). A *P* value less than 0.05 was considered to indicate statistical significance. All experiments were repeated three times.

## Results

### hsa_circ_0043278 was downregulated in BC tissues and cell lines

**Fig. 1:**
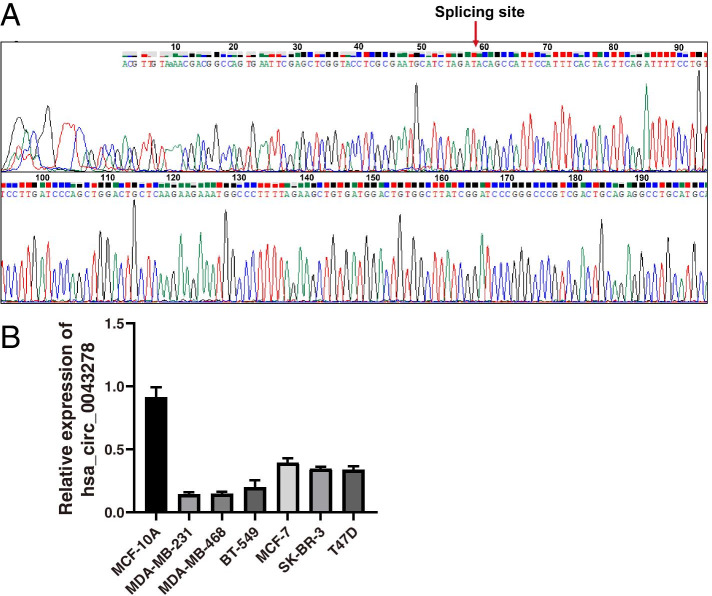
Hsa_circ_0043278 was downregulated in BC tissues and cell lines. **A** The PCR products of hsa_circ_0043278 were verified by DNA sequencing. The red arrow shows the back-splice junction site of hsa_circ_0043278. **B** The levels of hsa_circ_0043278 in MCF-10A cells and BC cell lines were determined by qRT–PCR. The data are presented as the mean ± standard deviation values (*n* = 3)

In our previous study, hsa_circ_0043278 was found to be downregulated 43-fold in BC tissues compared to matched normal tissues by microarray analysis. In addition, hsa_circ_0043278 was found to be downregulated in BC tissues compared to matched normal tissues by qRT–PCR [19]. In this study, BC cell lines (MDA-MB-231, MDA-MB-468, BT-549, MCF-7, SK-BR-3, and T47D) and MCF-10A normal breast epithelial cells were used to verify the differential expression of hsa_circ_0043278. The cDNA of hsa_circ_0043278 was amplified from HEK 293T cells and confirmed by DNA sequencing (Fig. 1A). As shown in Fig. 1B, the expression of hsa_circ_0043278 in BC cell lines was lower than that in the normal breast epithelial cell line MCF-10A. Moreover, hsa_circ_0043278 expression in TNBC cell lines (MDA-MB-231, MDA-MB-468, and BT-549) was much lower than that in cell lines of other subtypes of BC (MCF-7, SK-BR-3, and T47D). Thus, we chose MDA-MB-231 and MCF-7 cells, which had the lowest and highest expression of hsa_circ_0043278 among the BC cell lines, respectively, for our next study.

### hsa_circ_0043278 inhibits BC cell viability, migration and invasion

**Fig. 2:**
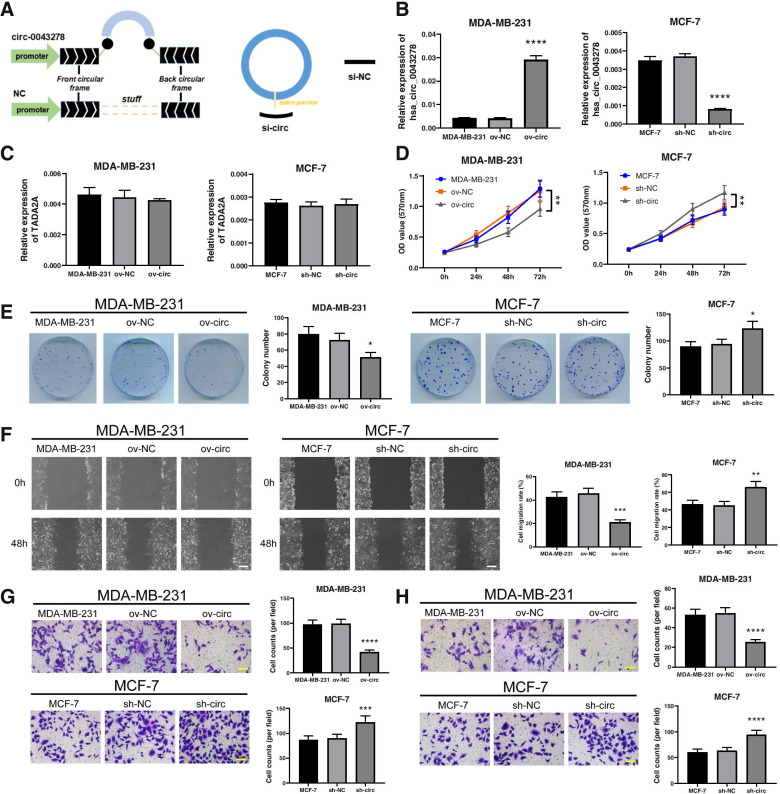
Hsa_circ_0043278 decreased BC cell viability and inhibited their migration and invasion. **A** Schematic illustration of the overexpression vector and siRNA of hsa_circ_0043278. The expression of (**B**) hsa_circ_0043278 and (**C**) TADA2A was verified by qRT–PCR in MDA-MB-231 and MCF-7 cells after upregulation or downregulation of hsa_circ_0043278, respectively. **D** The growth of MDA-MB-231 and MCF-7 cells in each group was analysed by MTT assays. **E** The viability of MDA-MB-231 and MCF-7 cells in each group was evaluated by colony formation assays. **F** The migration capacities of MDA-MB-231 and MCF-7 cells in each group were examined by wound healing assays. Scale bar, 200 μm. **G** The migration and (**H**) invasion abilities of MDA-MB-231 and MCF-7 cells in each group were analysed by Transwell assays. Scale bar, 100 μm. The data are presented as the mean ± standard deviation values (*n* = 3); * *P* < 0.05, ** *P* < 0.01, *** *P* < 0.001, **** *P* < 0.0001

To examine the biological role of hsa_circ_0043278 in BC cells, an hsa_circ_0043278 overexpression vector and a shRNA vector targeting hsa_circ_0043278 were constructed (Fig. 2A). Next, we upregulated hsa_circ_0043278 in MDA-MB-231 cells and downregulated it in MCF-7 cells. The expression of hsa_circ_0043278 was examined by qRT–PCR (Fig. 2B). Since hsa_circ_0043278 is spliced from the transcriptional adapter 2-alpha (TADA2A) gene (chr17, 35766977–35839830), the expression of the linear transcript TADA2A was also verified by qRT–PCR. The results demonstrated that there was no effect on the expression of TADA2A when hsa_circ_0043278 was overexpressed or knocked down in BC cells (Fig. 2C). Moreover, MTT assays were performed to examine the influence of hsa_circ_0043278 on the viability of BC cells. The results showed that upregulation of hsa_circ_0043278 markedly decreased the viability of MDA-MB-231 cells at 72 h (*P*<0.01), whereas downregulation of hsa_circ_0043278 significantly increased the viability of MCF-7 cells (*P*<0.01; Fig. 2D). In addition, the colony-forming ability of MDA-MB-231 cells was markedly reduced after upregulation of hsa_circ_0043278 (*P*<0.05) and was significantly increased in MCF-7 cells by downregulation of hsa_circ_0043278 (*P*<0.05; Fig. 2E). To explore the effects of hsa_circ_0043278 on cell migration and invasion, wound healing assays and Transwell assays were performed on BC cells. As shown in Fig. 2F, the wound healing assays revealed that the migration rate of MDA-MB-231 cells was markedly reduced after upregulation of hsa_circ_0043278 (*P*<0.001). Conversely, the migration rate of MCF-7 cells was markedly increased by downregulation of hsa_circ_0043278 (*P*<0.01). Similarly, the results of the Transwell assays showed that upregulation of hsa_circ_0043278 significantly inhibited the migration (Fig. 2G) and invasion (Fig. 2H) of MDA-MB-231 cells, while downregulation of hsa_circ_0043278 in MCF-7 cells resulted in the opposite effects (Fig. 2G-H). These results suggested that hsa_circ_0043278 decreased the viability and inhibited the migration and invasion of BC cells *in vitro*.

### Hsa_circ_0043278 inhibits the tumorigenesis of BC cells *in vivo*

**Fig. 3:**
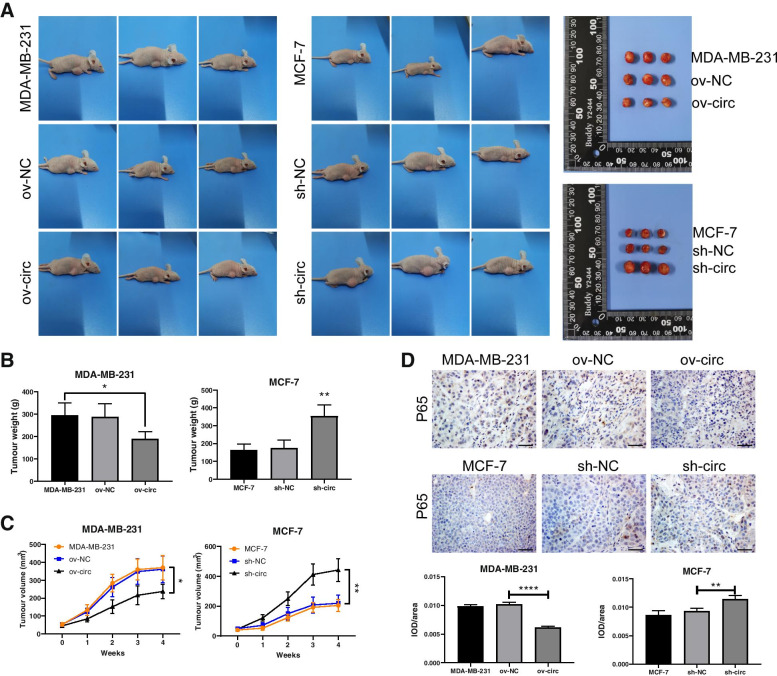
Hsa_circ_0043278 inhibited the tumorigenesis of BC cells *in vivo*. **A** Images of xenograft tumours from BALB/c nude mice in each group after injection of MDA-MB-231 cells with overexpression of hsa_circ_0043278 or MCF-7 cells with downregulation of hsa_circ_0043278. **B** The tumour weights were measured in each group. **C** Tumour growth in each group was monitored weekly after injection of transfected cells. **D** The protein levels of NF-κB (P65) in tumours were examined by immunohistochemical staining, and the corresponding quantification of nuclear P65 was performed. Scale bar, 50 μm. The data are presented as the mean ± standard deviation values (*n* = 3); * *P* < 0.05, ** *P* < 0.01, **** *P* < 0.0001. IOD, integrated optical density

To explore the role of hsa_circ_0043278 in tumorigenesis *in vivo*, we subcutaneously injected MDA-MB-231 cells transfected with the hsa_circ_0043278 overexpression vector (ov-circ) or mock vector (ov-NC) or MCF-7 cells transfected with hsa_circ_0043278 shRNA (sh-circ) or the negative control shRNA (sh-NC) into female nude mice. After 4 weeks, the tumours formed from ov-circ MDA-MB-231 cells were significantly smaller and lighter than the tumours formed in the control group (*P*<0.05), and the tumours formed from MCF-7 cells transfected with sh-circ were larger and heavier than the tumours formed in the sh-NC group (*P*<0.01; Fig. 3A-B). Moreover, the tumour volume in the ov-circ group increased at a significantly lower rate than that in the ov-NC group every week, and the tumours in the ov-circ group were significantly smaller than those in the ov-NC group at the 4th week (*P*<0.05). Conversely, downregulation of hsa_circ_0043278 significantly promoted tumour growth (*P*<0.01; Fig. 3C). Furthermore, NF-κB (P65) is considered to play an important role in promoting tumour progression and invasion [22]. In the canonical pathway, NF-κB is translocated to the nucleus and activated after IκBα-mediated phosphorylation in an IKKβ- and NEMO-dependent manner [23]. In addition, NF-κB has been reported to be inhibited by EI24 in BC cells [21]. Thus, inhibition of nuclear NF-κB might be effective for the inhibition of BC progression. To determine the influence of hsa_circ_0043278 on P65, immunohistochemical staining of tumour tissues was performed. The results showed that upregulation of hsa_circ_0043278 notably reduced the expression of P65 in the nucleus (*P*<0.0001); in contrast, downregulation of hsa_circ_0043278 significantly increased the level of P65 in xenograft tumour tissues (*P*<0.01; Fig. 3D). These results indicated that hsa_circ_0043278 might inhibit tumour growth and affect NF-κB activation *in vivo*.

### Hsa_circ_0043278 functions as a sponge for miR-455-3p

**Fig. 4:**
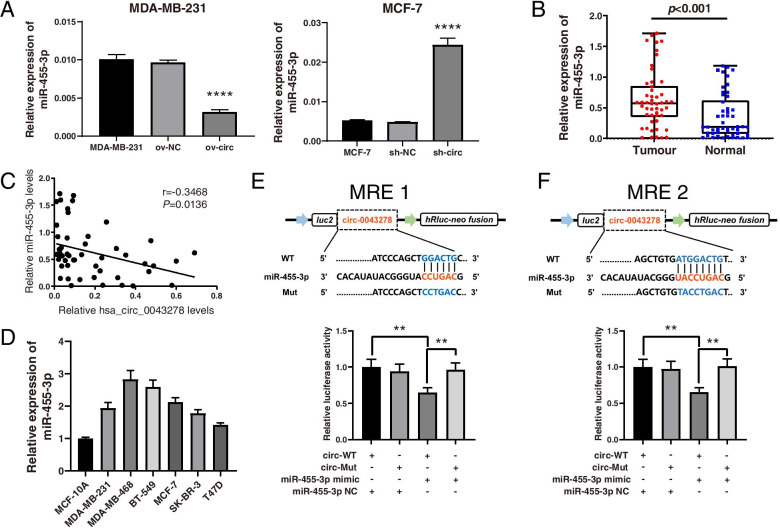
Hsa_circ_0043278 regulated and functioned as a sponge of miR-455-3p. **A** qRT–PCR analysis of miR-455-3p expression in MDA-MB-231 cells and MCF-7 cells with upregulation or downregulation of hsa_circ_0043278. **B** Verification of the relative expression of miR-455-3p in BC tissues (Tumour) and matched normal tissues (Normal) by qRT–PCR (*n* = 50). The middle horizontal lines in the scatter plot indicate the medians. **C** Pearson correlation analysis of miR-455-3p and hsa_circ_0043278 expression in BC tissues (*n* = 50). **D** The relative expression of miR-455-3p in cell lines was analysed by qRT–PCR. **E** and **F** Schematic illustration of the hsa_circ_0043278-WT and hsa_circ_0043278 mutant (Mut) luciferase reporter vectors with mutation of the two predicted binding sites of miR-455-3p. After transfection with the hsa_circ_0043278-WT (circ-WT) or hsa_circ_0043278-Mut (circ-Mut) vector and the miR-455-3p mimic or miR-455-3p NC into HEK 293T cells, relative luciferase activities were measured in each group. The data are presented as the mean ± standard deviation values (*n* = 3); ** *P* < 0.01, **** *P* < 0.0001. WT, wild-type; NC, negative control

In our previous study, we predicted the 5 miRNAs most likely to interact with hsa_circ_0043278: miR-455-3p, miR-103a-2-5p, miR-302b-3p, miR-302c-3p, and miR-520d-3p [19]. To explore the molecular mechanism of hsa_circ_0043278 in BC, qRT–PCR was performed to examine the expression levels of miRNAs after upregulation or downregulation of hsa_circ_0043278 in BC cells. Among the 5 abovementioned miRNAs, miR-455-3p exhibited markedly decreased expression compared to that in the control group when hsa_circ_0043278 was overexpressed in MDA-MB-231 cells (*P*<0.0001), whereas its expression was significantly elevated after knockdown of hsa_circ_0043278 in MCF-7 cells (*P*<0.0001; Fig. 4A). The levels of the other 4 predicted miRNAs showed no obvious changes after upregulation of hsa_circ_0043278 in MDA-MB-231 cells (Fig. S2). Thus, miR-455-3p might be a target of hsa_circ_0043278 in BC cells. Furthermore, miR-455-3p was determined to be markedly upregulated in 50 BC tissues compared with the matched normal tissues by qRT–PCR (*P*<0.001; Fig. 4B). Additionally, Pearson correlation analysis of these 50 BC tissue samples showed a significant negative correlation between the levels of miR-455-3p and hsa_circ_0043278 (*r*=-0.3468, *P*=0.0136; Fig. 4C). Similarly, the level of miR-455-3p was elevated in all BC cell lines compared to MCF-10A cells, as determined by qRT–PCR (Fig. 4D). Thus, to determine whether hsa_circ_0043278 serves as a competing endogenous RNA (ceRNA) for miR-455-3p, a dual-luciferase reporter assay was performed in HEK 293T cells. Bioinformatics prediction analysis showed that there were two potential binding sites between miR-455-3p and hsa_circ_0043278 [19]. We subcloned the full-length wild-type hsa_circ_0043278 sequence (circ-WT) and two mutant versions without miR-455-3p binding sites (circ-Mut) into luciferase reporter vectors (Fig. 4E-F). We found that in the circ-WT groups, transfection with the miR-455-3p mimics markedly reduced the luciferase activity compared to that in the miR-455-3p NC group (*P*<0.01). Moreover, the miR-455-3p mimics did not decrease the luciferase activity in either circ-Mut group (Fig. 4E-F). This suggested that hsa_circ_0043278 might function as a sponge for miR-455-3p through the two predicted binding sites.

### EI24 is a target of miR-455-3p and is indirectly regulated by hsa_circ_0043278

**Fig. 5:**
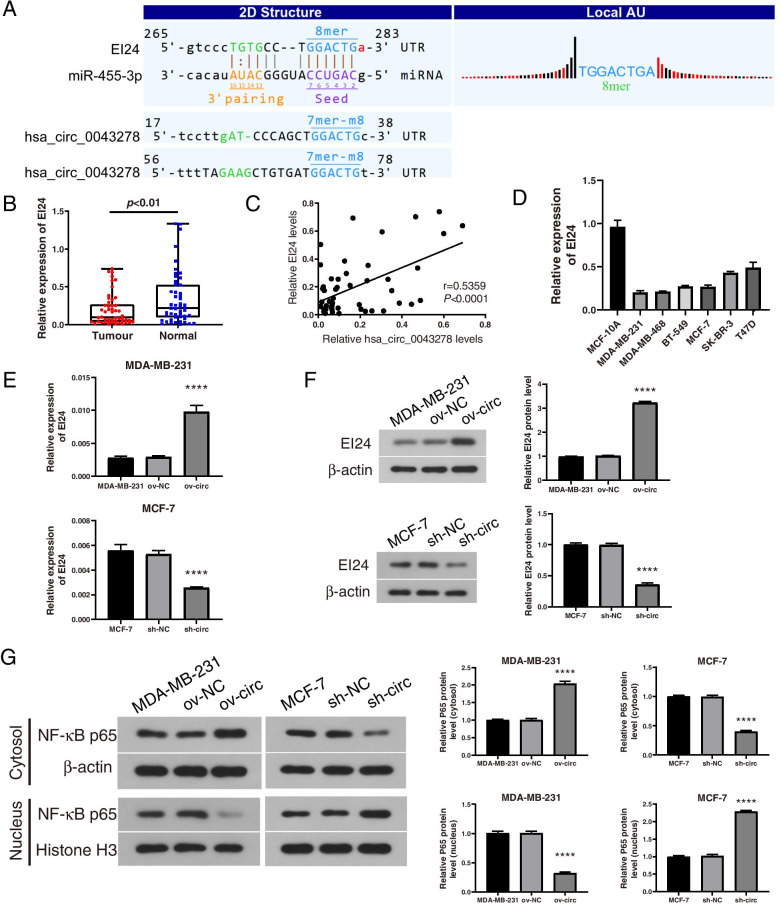
EI24 was directly targeted by miR-455-3p and regulated by hsa_circ_0043278. **A** Prediction of miR-455-3p binding sites in EI24 and hsa_circ_0043278 using TargetScan and MiRanda. The binding sites are presented as solid lines. **B** Verification of the relative expression of EI24 in BC tissues (Tumour) and matched normal tissues (Normal) by qRT–PCR (*n* = 50). The middle horizontal lines in the scatter plot indicate the medians. **C** Pearson correlation analysis of EI24 and hsa_circ_0043278 expression in BC tissues (*n* = 50). **D** Relative mRNA levels of EI24 in cell lines were determined by qRT–PCR. **E** qRT–PCR and (**F**) western blot analysis of the EI24 mRNA level in MDA-MB-231 cells and MCF-7 cells after upregulation or downregulation of hsa_circ_0043278. (The images of the western blot bands were cropped from the images shown in Figs. S3 and S4). **G** The relative protein level of NF-κB (P65) in the cytosol and nucleus of cells in each group were determined by western blot analysis. (The images of the western blot bands were cropped from the images shown in Figs. S5, S6, S7, and S8.) The data are presented as the mean ± standard deviation values (*n* = 3); **** *P* < 0.0001

To explore the mechanism of hsa_circ_0043278 in BC, we predicted the downstream targets of miR-455-3p. In our previous study, two potential binding sites between miR-455-3p and hsa_circ_0043278 were predicted [19]. According to the TargetScan and miRanda prediction results in this study, EI24 and hsa_circ_0043278 were most likely to share the same miRNA response element (MRE) for miR-455-3p (Fig. 5A). In addition, EI24 was reported to be a direct target of miR-455-3p and to act as a tumour suppressor gene in triple-negative BC (TNBC) [20]. Thus, we hypothesized that EI24 might be indirectly regulated by hsa_circ_0043278 through miR-455-3p. Furthermore, EI24 expression was verified to be decreased in the 50 BC tissues compared to the matched normal tissues by qRT–PCR (*P*<0.01; Fig. 5B). Moreover, the expression level of EI24 was positively associated with the hsa_circ_0043278 expression level in Pearson correlation analysis (*r*=0.5359, *P*<0.0001; Fig. 5C). The expression of EI24 was also decreased in BC cell lines compared to MCF-10A cells (Fig. 5D). After verifying that hsa_circ_0043278 can directly bind to miR-455-3p, we then examined whether hsa_circ_0043278 affects EI24 expression to understand the role of the hsa_circ_0043278/miR-455-3p/EI24 axis in BC cells. The qRT–PCR results indicated that overexpression of hsa_circ_0043278 significantly increased the level of EI24 in MDA-MB-231 cells (*P*<0.0001) and that knockdown of hsa_circ_0043278 markedly decreased the level of EI24 in MCF-7 cells (*P*<0.0001; Fig. 5E). Similarly, western blot analysis indicated that the protein level of EI24 was also positively regulated by hsa_circ_0043278 in the 2 BC cell lines (*P*<0.0001; Fig. 5F). These results showed that hsa_circ_0043278 might regulate the expression level of EI24 by functioning as a sponge for miR-455-3p in BC.

Next, we examined whether hsa_circ_0043278 affects the expression of P65 in the nucleus of BC cells. Western blot analysis suggested that upregulation of hsa_circ_0043278 markedly decreased the protein level of P65 in the nucleus (*P*<0.0001) and increased it in the cytosol in MDA-MB-231 cells (*P*<0.0001), while knockdown of hsa_circ_0043278 resulted in the opposite effects in MCF-7 cells (Fig. 5G). These results revealed that hsa_circ_0043278 influenced the expression of the downstream gene of EI24 and might act as a tumour suppressor by inhibiting NF-κB in BC cells.

### Validation of hsa_circ_0043278-mediated inhibition of BC progression through the hsa_circ_0043278/miR-455-3p/EI24 axis

**Fig. 6:**
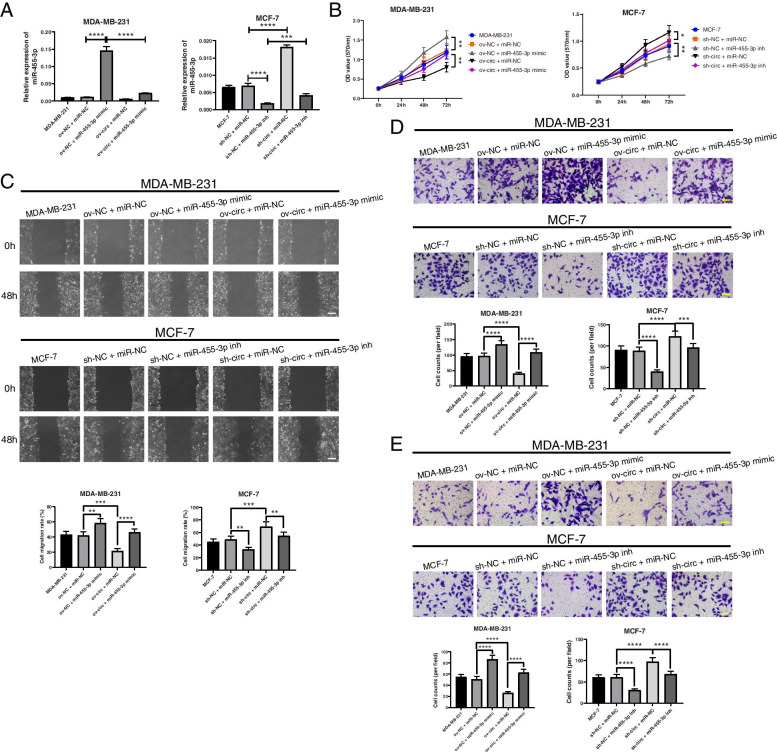
Hsa_circ_0043278 inhibits BC cell growth, migration and invasion by sponging miR-455-3p. **A** qRT–PCR was used to examine the relative expression of miR-455-3p in MDA-MB-231 cells after transfection of the hsa_circ_0043278 overexpression vector and the miR-455-3p mimic, as well as in MCF-7 cells after transfection of hsa_circ_0043278 shRNA and the miR-455-3p inhibitor. **B** The growth of MDA-MB-231 and MCF-7 cells in each group was analysed by MTT assays. **C** The migration capacities of MDA-MB-231 and MCF-7 cells in each group were examined by wound healing assays. Scale bar, 200 μm. **D** and **E** The migration and invasion abilities of MDA-MB-231 and MCF-7 cells in each group were analysed by Transwell assays. Scale bar, 100 μm. The data are presented as the mean ± standard deviation values (*n* = 3); * *P* < 0.05, ** *P* < 0.01, *** *P* < 0.001, **** *P* < 0.0001

After verifying that hsa_circ_0043278 directly binds to miR-455-3p and regulates the EI24 mRNA and protein levels, it was essential to explore whether hsa_circ_0043278 acts in BC progression through the hsa_circ_0043278/miR-455-3p/EI24 axis. miR-455-3p mimics and inhibitors were designed and cotransfected into MDA-MB-231 and MCF-7 cells. After that, the level of miR-455-3p was examined by qRT–PCR (Fig. 6A). The MTT assay showed that upregulation of hsa_circ_0043278 significantly decreased the viability of MDA-MB-231 cells and that this effect could be suppressed by cotransfection of the miR-455-3p mimic. Furthermore, downregulation of hsa_circ_0043278 significantly increased the viability of MCF-7 cells, but this effect was abolished by cotransfection of the miR-455-3p inhibitor (Fig. 6B). Additionally, the wound healing assays demonstrated that the miR-455-3p mimic counteracted the reduction in the migration rate of MDA-MB-231 cells induced by overexpression of hsa_circ_0043278, while the miR-455-3p inhibitor reversed the increase in the migration rate of MCF-7 cells induced by silencing hsa_circ_0043278 (Fig. 6C). Similarly, the Transwell assays revealed that the influences of upregulating or silencing hsa_circ_0043278 on BC cell migration and invasion were rescued by the miR-455-3p mimic and inhibitor, respectively (Fig. 6D-E).

**Fig. 7:**
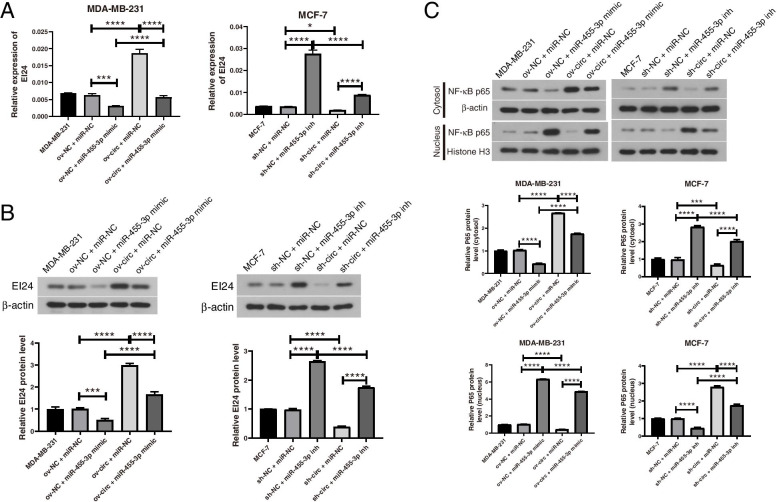
Hsa_circ_0043278 regulates EI24 and NF-κB expression by sponging miR-455-3p. **A** qRT–PCR and **B** western blotting were used to examine the relative expression of EI24 in MDA-MB-231 cells after transfection of the hsa_circ_0043278 overexpression vector and the miR-455-3p mimic as well as in MCF-7 cells after transfection of hsa_circ_0043278 shRNA and the miR-455-3p inhibitor. (The images of the western blot bands were cropped from the images shown in Figs. S9 and S10). **C** Relative protein levels of NF-κB (P65) in the cytosol or nucleus of MDA-MB-231 and MCF-7 cells in each group were determined by western blot analysis. (The images of the western blot bands were cropped from the images shown in Figs. S11, S12, S13, and S14.) The data are presented as the mean ± standard deviation values (*n* = 3); * *P* < 0.05, *** *P* < 0.001, **** *P* < 0.0001

Subsequently, the qRT–PCR and western blot results revealed that upregulation of hsa_circ_0043278 increased the mRNA and protein levels of EI24 in MDA-MB-231 cells, while downregulation of hsa_circ_0043278 decreased the mRNA and protein levels of EI24 in MCF-7 cells. These effects were reversed by cotransfection of the miR-455-3p mimic or inhibitor (Fig. 7A-B). In addition, the level of P65 was significantly decreased in the nucleus after overexpression of hsa_circ_0043278 and increased by downregulation of hsa_circ_0043278; these effects were also counteracted by the miR-455-3p mimic or inhibitor (Fig. 7C). These results showed that hsa_circ_0043278 might function through the hsa_circ_0043278/miR-455-3p/EI24 axis to regulate the development of BC.

## Discussion

BC represents the most common malignancy and the leading cause of cancer death in women worldwide [24]. Thus, finding a new therapeutic target is an urgent need to improve the survival of BC patients. Sequencing technology has shown that circRNAs are involved in various malignancies and function as diagnostic markers or therapeutic targets [8, 25]. circRNAs are recognized as a special class of RNA and are increasingly becoming a hotspot of RNA research. However, our knowledge of circRNAs is still limited [25, 26]. The relationship between circRNAs and BC has rarely been reported. Discovering the biological roles of circRNAs could be beneficial for the diagnosis and treatment of BC.

Previously, we found that hsa_circ_0043278 was significantly downregulated in BC tissues. The downregulated expression of hsa_circ_0043278 in BC cell lines relative to normal mammary epithelial cells was verified. These findings led us to discover the correlation between hsa_circ_0043278 and BC. Thus, we selected MCF-7 (with the highest expression of hsa_circ_0043278) and MDA-MB-231 (with the lowest expression of hsa_circ_0043278) cells for subsequent research. Functional experiments suggested that overexpression of hsa_circ_0043278 significantly decreased the viability and the migration and invasion abilities of BC cells *in vitro* and decreased tumour growth *in vivo*, while downregulation of hsa_circ_0043278 showed the opposite effects. These results suggested that hsa_circ_0043278 might act as a tumour suppressor gene in BC development.

miRNAs have been reported to bind to the 3'UTR and inhibit the expression of their target mRNAs [27–31]. CircRNAs have been generally reported to act as potent miRNA sponges [12] via MREs, thus inhibiting miRNA expression [32, 33]. In our previous study, we predicted that hsa_circ_0043278 contained 2 possible MREs for miR-455-3p [19]. In addition, miR-455-3p has been reported to regulate EI24 expression in TNBC and to serve as an oncogene [20]. Thus, we speculated that hsa_circ_0043278 might function by targeting miR-455-3p and regulating EI24 in BC. In our current study, miR-455-3p was found to be overexpressed in BC tissues and cell lines. The expression of miR-455-3p was negatively correlated with that of hsa_circ_0043278 in BC tissues. Moreover, the qRT-PCR and dual-luciferase reporter assay results suggested that hsa_circ_0043278 suppressed the expression of miR-455-3p by directly sponging it. The results proved the relationship between hsa_circ_0043278 and miR-455-3p in BC.

Furthermore, EI24 has been reported to be a target gene of miR-455-3p [20]. It is also considered to act a tumour suppressor gene by inhibiting NF-κB activity [21]. In the current study, EI24 and hsa_circ_0043278 were the most likely circRNA-miRNA target pair to share the same MRE for miR-455-3p. Next, we confirmed that EI24 was also markedly downregulated in BC tissues and cell lines. Our results showed that overexpression of hsa_circ_0043278 increased the EI24 level and suppressed the activity of NF-κB in BC cell lines. The rescue experiments demonstrated that inhibiting miR-455-3p partially suppressed the tumour-enhancing effect induced by silencing of hsa_circ_0043278 in MCF-7 cells. The changes in the level of EI24 and the activity of NF-κB were also partially reversed. Moreover, enhancing miR-455-3p expression showed the opposite effect in MDA-MB-231 cells. These results supported the hypothesis that hsa_circ_0043278 acts as a ceRNA of miR-455-3p to decrease the viability and inhibit the migration and invasion of BC cells. The hsa_circ_0043278/miR-455-3p/EI24 signalling axis might facilitate the progression of BC.

Additionally, we found that hsa_circ_0043278 expression was much lower in TNBC cell lines than in cell lines of other subtypes of BC. Thus, the relationship between TNBC and hsa_circ_0043278 needs to be verified in the future. The precise relationship between hsa_circ_0043278 and NF-κB also needs to be elucidated. Furthermore, the number of patient samples needs to be increased. Multicentre trials are also needed to investigate the roles of hsa_circ_0043278.

## Conclusions

This study identified downregulation of hsa_circ_0043278 in BC cells and tissues. Additionally, hsa_circ_0043278 was found to significantly inhibit BC progression by sponging miR-455-3p, thus influencing the level of EI24 and the activity of NF-κB. Hsa_circ_0043278 may be regarded as a novel prognostic predictor and a potential therapeutic target for BC. The hsa_circ_0043278/miR-455-3p/EI24 axis might be a crucial mechanism of BC cell progression.

## Supplementary Information

**Additional file 1: Table S1.** The sequences of primers in this study

**Additional file 2: Table S2.** The sequences of siRNAs and shRNAs

**Additional file 3: Table S3.** miR-455-3p inhibitor sequence

**Additional file 4: Figure S1.** Two siRNAs (siRNA-1 and siRNA-2) targeting the back-splice junction site of hsa_circ_0043278 were constructed, and the expression of hsa_circ_0043278 in MCF-7 cells was analysed by qRT–PCR. The data are presented as the mean ± standard deviation values (*n* = 3); **** *P* < 0.0001.

**Additional file 5: Figure S2.** The expression of miR-103a-2-5p, miR-302b-3p, miR-302c-3p, and miR-520d-3p was measured by qRT–PCR after overexpression of hsa_circ_0043278 in MDA-MB-231 cells. The data are presented as the mean ± standard (*n* = 3).

**Additional file 6: Figure S3.** Western blot bands of EI24 (A) and β-actin (B) after hsa_circ_0043278 overexpression in MDA-MB-231 cells.

**Additional file 7: Figure S4.** Western blot bands of β-actin (A) and EI24 (B) after hsa_circ_0043278 downregulation in MCF-7 cells.

**Additional file 8: Figure S5.** Western blot bands of NF-κB (P65) (A) and β-actin (B) in the cytosol after hsa_circ_0043278 overexpression in MDA-MB-231 cells.

**Additional file 9: Figure S6.** Western blot bands of histone H3 (A) and NF-κB (P65) (B) in the nucleus after hsa_circ_0043278 overexpression in MDA-MB-231 cells.

**Additional file 10: Figure S7.** Western blot bands of β-actin (A) and NF-κB (P65) (B) in the cytosol after silencing hsa_circ_0043278 in MCF-7 cells.

**Additional file 11: Figure S8.** Western blot bands of histone H3 (A) and NF-κB (P65) (B) in the nucleus after silencing hsa_circ_0043278 in MCF-7 cells.

**Additional file 12: Figure S9.** Western blot bands of EI24 (A) and β-actin (B) after hsa_circ_0043278 overexpression and/or transfection of the miR-455-3p mimic in MDA-MB-231 cells.

**Additional file 13: Figure S10.** Western blot bands of β-actin (A) and EI24 (B) after hsa_circ_0043278 downregulation and/or transfection of the miR-455-3p inhibitor in MCF-7 cells.

**Additional file 14: Figure S11.** Western blot bands of NF-κB (P65) (A) and β-actin (B) in the cytosol after hsa_circ_0043278 overexpression and/or transfection of the miR-455-3p mimic in MDA-MB-231 cells.

**Additional file 15: Figure S12.** Western blot bands of histone H3 (A) and NF-κB (P65) (B) in the nucleus after hsa_circ_0043278 overexpression and/or transfection of the miR-455-3p mimic in MDA-MB-231 cells.

**Additional file 16: Figure S13.** Western blot bands of NF-κB (P65) (A) and β-actin (B) in the cytosol after hsa_circ_0043278 downregulation and/or transfection of the miR-455-3p inhibitor in MCF-7 cells.

**Additional file 17: Figure S14.** Western blot bands of histone H3 (A) and NF-κB (P65) (B) in the nucleus after hsa_circ_0043278 downregulation and/or transfection of the miR-455-3p inhibitor in MCF-7 cells.

## Data Availability

The datasets used during the current study are available from the corresponding author on reasonable request.

## Abbreviations

BC: Breast cancer
circRNA: Circular RNA
miRNA: microRNA
3'UTR: 3′-untranslated region
ceRNA: Competing endogenous RNA
MRE: miRNA response element
IHC: Immunohistochemistry
