# Supplementary material for: Circular RNA hsa_circ_0043278 inhibits breast cancer progression via the miR-455-3p/EI24 signalling pathway
Source: BMC Cancer. 2021 Nov 20;21:1249. doi: 10.1186/s12885-021-08989-w (PMC8605514; doi:10.1186/s12885-021-08989-w)
Supplement: Supplementary file 1 — Additional file 1: Table S1. The sequences of primers in this study [file 12885_2021_8989_MOESM1_ESM.pdf]

**Circular RNA hsa_circ_0043278 Inhibits Breast Cancer Cell Progression via miR-455-3p/EI24 Signal Pathway**

**Yue Shi [1] and Chong Liu [2]**

1 Department of Geriatric Surgery, The First Affiliated Hospital of China Medical University, Shenyang 110001, China

2 Department of Breast Surgery, The First Affiliated Hospital of China Medical University, Shenyang 110001, China

**Additional file 1 Table S1** The sequences of primers in this study

| RNA | Forward (5'-3') | Reverse (5'-3') |
|---|---|---|
| **hsa_circ_0043278** | ACAGCCATTCCATTTCAC | AAGCCACAGTCCATCACA |
| **EI24** | TTGAGGTATCAGGGAGGA | GACAAGATGGATGGGAAA |
| **TADA2A** | ATGGACTGTGGCTTTGGA | TTCAGGTTCAGCAGGGTA |
| **β-actin** | CGGGAAATCGTGCGTGAC | GTCAGGCAGCTCGTAGCTCTT |
| **miR-455-3p** | GCAGTCCATGGGCATATACAC | GCAGGGTCCGAGGTATTC |
| **U6** | GCTTCGGCAGCACATATACT | GTGCAGGGTCCGAGGTATTC |

Note: EI24 etoposide-induced gene 24, TADA2A Transcriptional adapter 2-alpha
