# Supplementary material for: Circular RNA hsa_circ_0043278 inhibits breast cancer progression via the miR-455-3p/EI24 signalling pathway
Source: BMC Cancer. 2021 Nov 20;21:1249. doi: 10.1186/s12885-021-08989-w (PMC8605514; doi:10.1186/s12885-021-08989-w)
Supplement: Supplementary file 2 — Additional file 2: Table S2. The sequences of siRNAs and shRNAs [file 12885_2021_8989_MOESM2_ESM.pdf]

**Circular RNA hsa_circ_0043278 Inhibits Breast Cancer Cell Progression via miR-455-3p/EI24 Signal Pathway**

**Yue Shi [1] and Chong Liu [2]**

1 Department of Geriatric Surgery, The First Affiliated Hospital of China Medical University, Shenyang 110001, China

2 Department of Breast Surgery, The First Affiliated Hospital of China Medical University, Shenyang 110001, China

**Additional file 2 Table S2** The sequences of siRNAs and shRNAs

| siRNAs or shRNAs | Sequence (5'-3') |
|---|---|
| **hsa-circ-0043278 siRNA-1** | AAGUAGUGAAAUGGAAUGGTT |
| **hsa-circ-0043278 siRNA-2** | AUCUGAAGUAGUGAAAUGGTT |
| **si-NC** | ACGUGACACGUUCGGAGAATT |
| **sh-circ** | GATCCGCCATTTCACTACTTCAGATTTCAAGAGAATC TGAAGTAGTGAAATGGTTTTTA |
| **sh-NC** | GATCCCCTTCTCCGAACGTGTCACGTTTCAAGAGAA CGTGACACGTTCGGAGAATTTTT |

Note: si-NC siRNA of the negative control, sh-circ shRNA of hsa-circ-0043278, sh-NC shRNA of the negative control
