# Supplementary material for: Circular RNA hsa_circ_0043278 inhibits breast cancer progression via the miR-455-3p/EI24 signalling pathway
Source: BMC Cancer. 2021 Nov 20;21:1249. doi: 10.1186/s12885-021-08989-w (PMC8605514; doi:10.1186/s12885-021-08989-w)
Supplement: Supplementary file 3 — Additional file 3: Table S3. miR-455-3p inhibitor sequence [file 12885_2021_8989_MOESM3_ESM.pdf]

Circular RNA hsa_circ_0043278 Inhibits Breast Cancer Cell Progression via miR-455-3p/EI24 Signal Pathway

Yue Shi [1] and Chong Liu [2]

1 Department of Geriatric Surgery, The First Affiliated Hospital of China Medical University, Shenyang 110001, China

2 Department of Breast Surgery, The First Affiliated Hospital of China Medical University, Shenyang 110001, China

Additional file 3 Table S3 miR-455-3p inhibitor sequence

Inhibitor	Sequence (5'-3')
**miR-455-3p inhibitor**	GUGUAUAUGCCCAUGGACUGC
**Inhibitor NC**	UUGUACUACACAAAAGUACUG

Note: NC negative control
