# Supplementary figures and images for: Circular RNA hsa_circ_0043278 inhibits breast cancer progression via the miR-455-3p/EI24 signalling pathway
Source: BMC Cancer. 2021 Nov 20;21:1249. doi: 10.1186/s12885-021-08989-w (PMC8605514; doi:10.1186/s12885-021-08989-w)

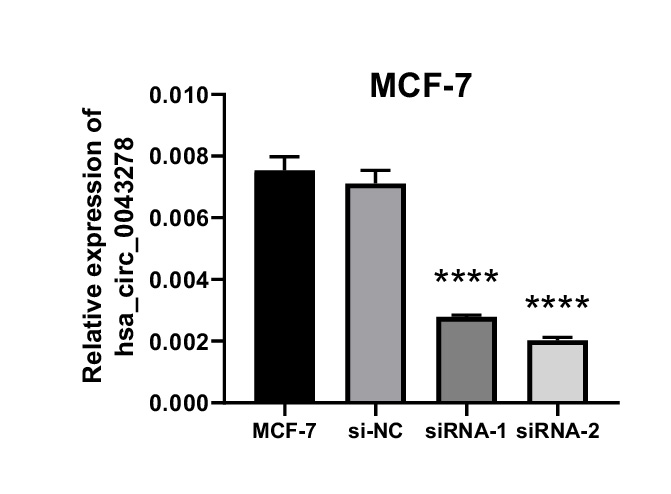

Supplement: Supplementary file 4 — Additional file 4: Figure S1. Two siRNAs (siRNA-1 and siRNA-2) targeting the back-splice junction site of hsa_circ_0043278 were constructed, and the expression of hsa_circ_0043278 in MCF-7 cells was analysed by qRT–PCR. The data are presented as the mean ± standard deviation values (n = 3); **** P < 0.0001. [file 12885_2021_8989_MOESM4_ESM.jpg]

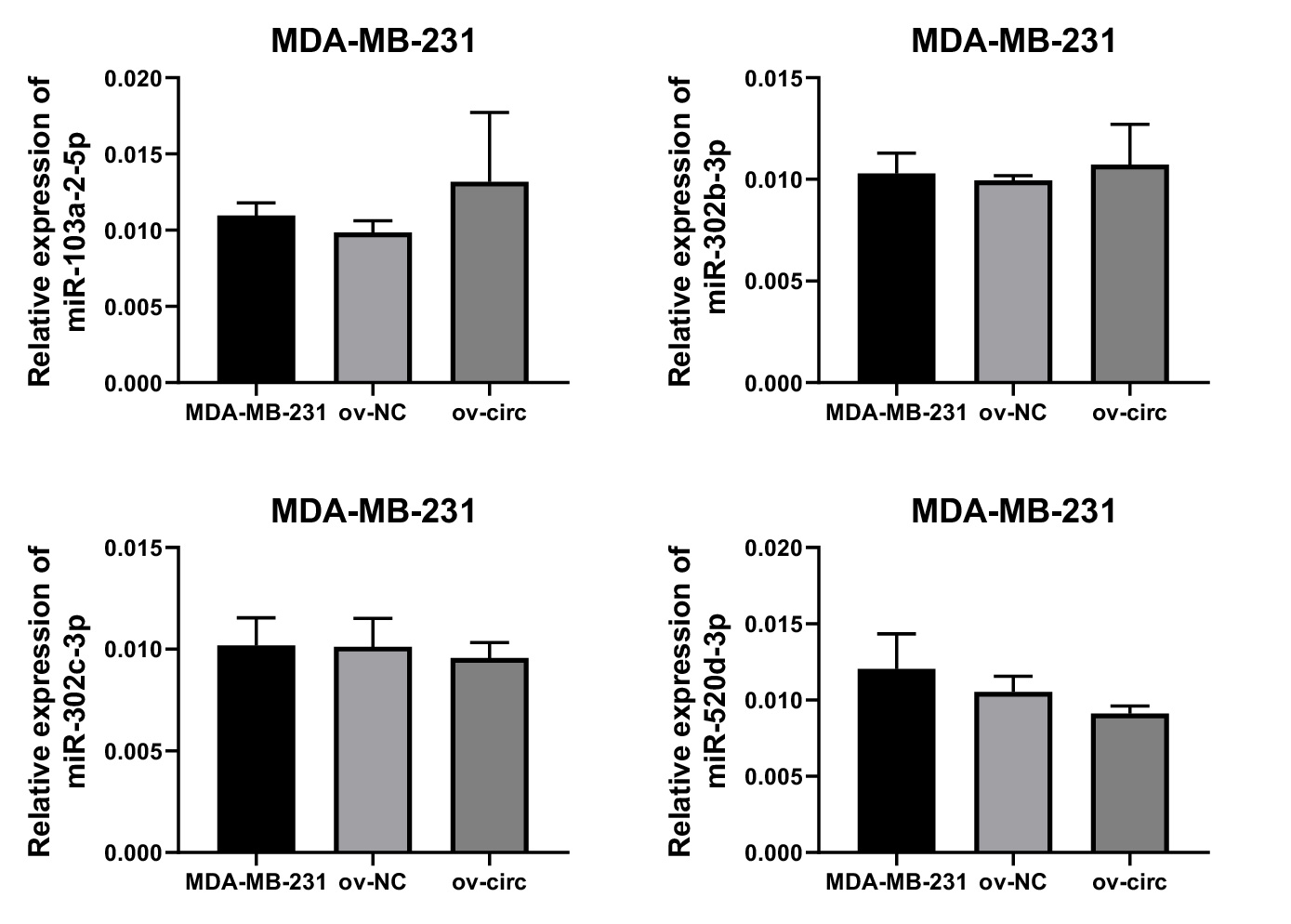

Supplement: Supplementary file 5 — Additional file 5: Figure S2. The expression of miR-103a-2-5p, miR-302b-3p, miR-302c-3p, and miR-520d-3p was measured by qRT–PCR after overexpression of hsa_circ_0043278 in MDA-MB-231 cells. The data are presented as the mean ± standard (n = 3). [file 12885_2021_8989_MOESM5_ESM.jpg]

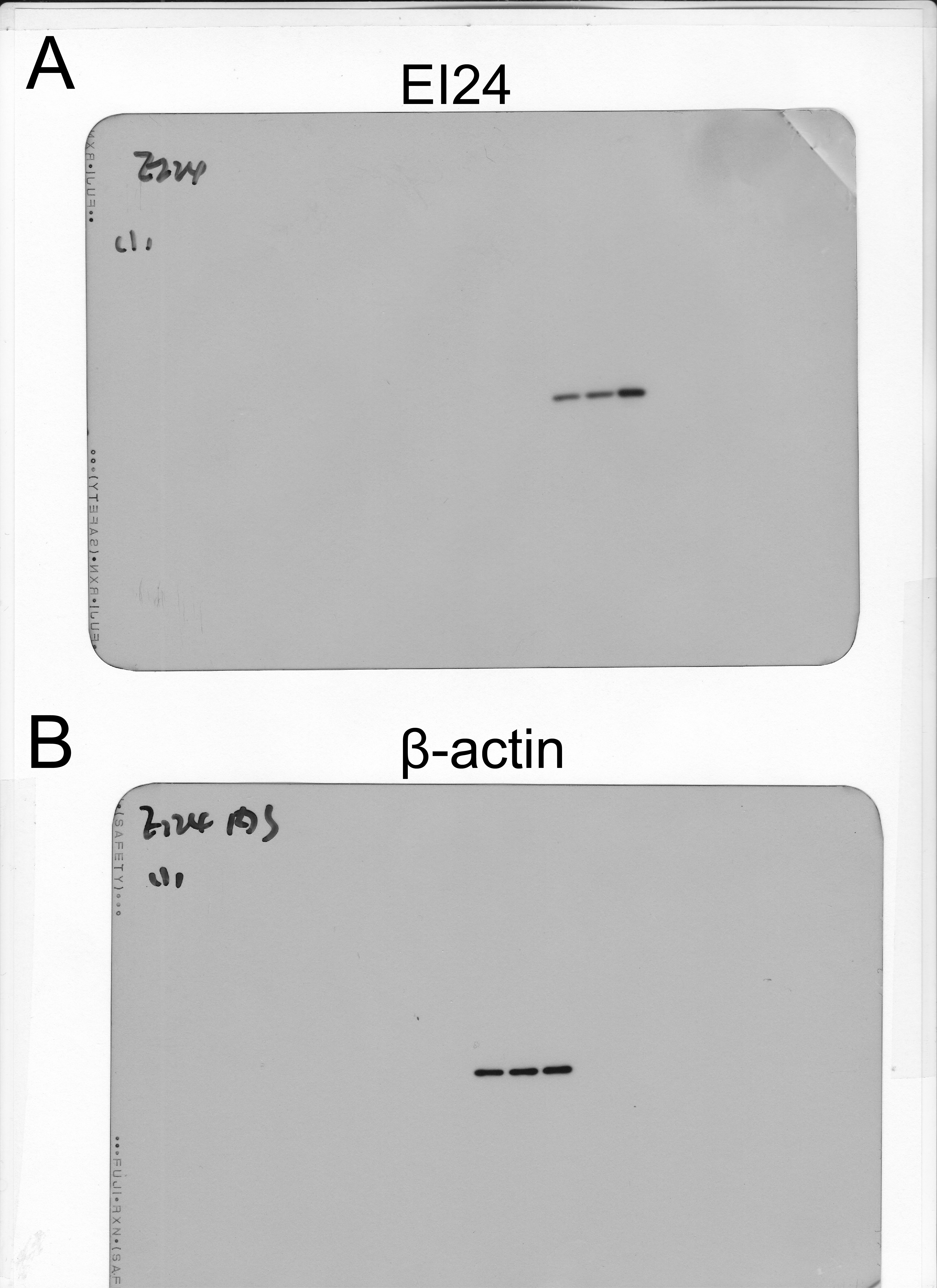

Supplement: Supplementary file 6 — Additional file 6: Figure S3. Western blot bands of EI24 (A) and β-actin (B) after hsa_circ_0043278 overexpression in MDA-MB-231 cells. [file 12885_2021_8989_MOESM6_ESM.jpg]

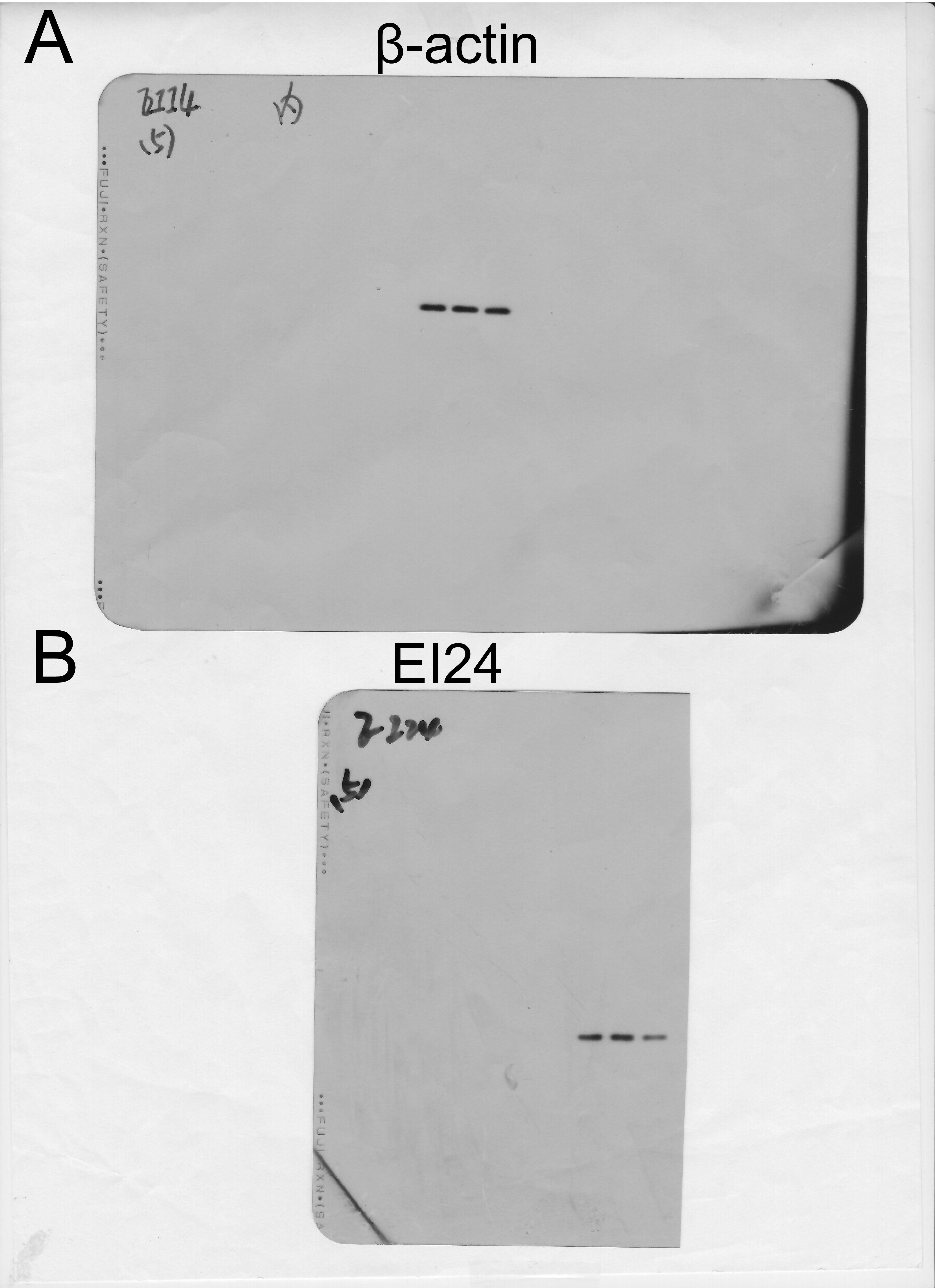

Supplement: Supplementary file 7 — Additional file 7: Figure S4. Western blot bands of β-actin (A) and EI24 (B) after hsa_circ_0043278 downregulation in MCF-7 cells. [file 12885_2021_8989_MOESM7_ESM.jpg]

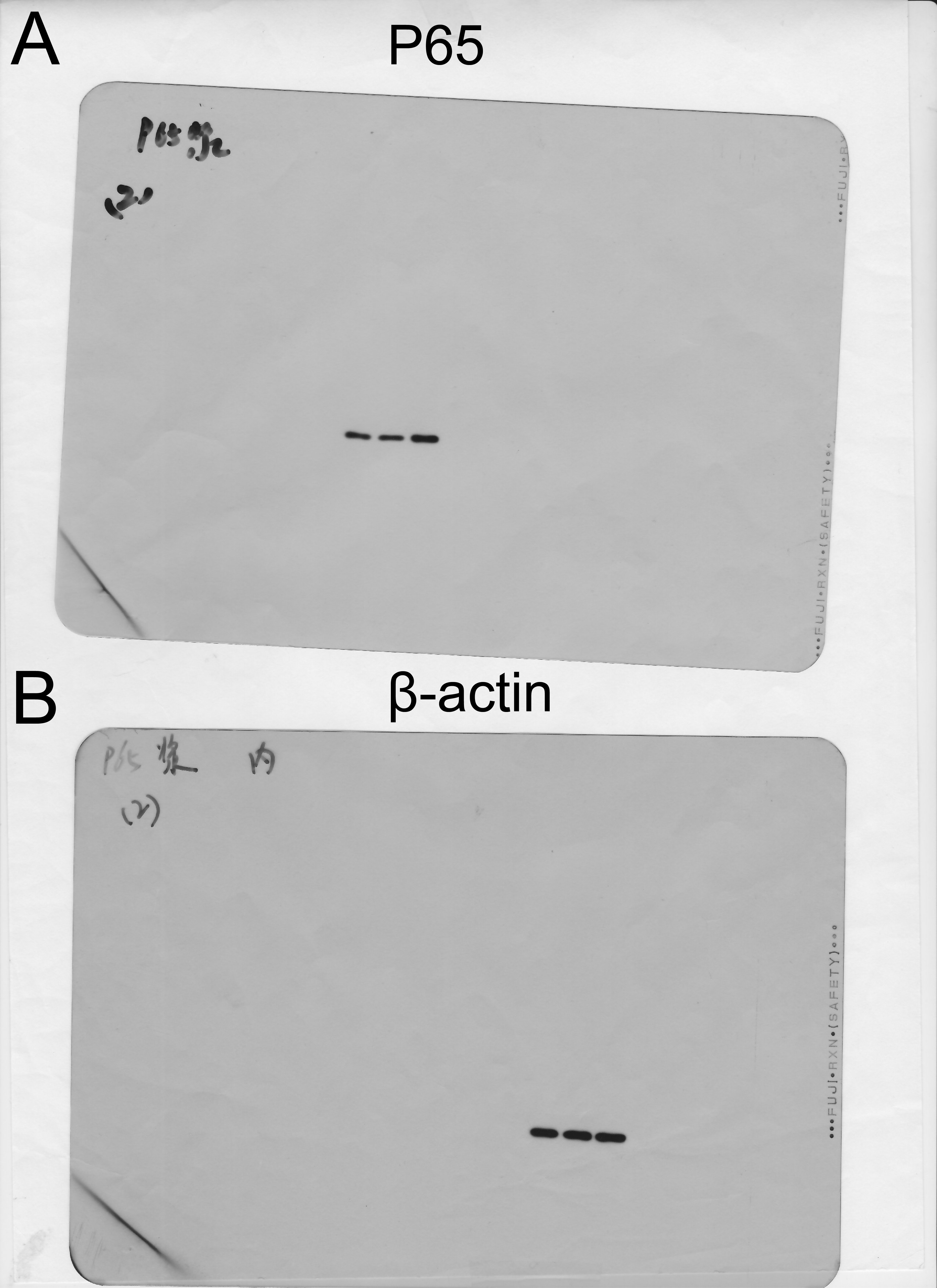

Supplement: Supplementary file 8 — Additional file 8: Figure S5. Western blot bands of NF-κB (P65) (A) and β-actin (B) in the cytosol after hsa_circ_0043278 overexpression in MDA-MB-231 cells. [file 12885_2021_8989_MOESM8_ESM.jpg]

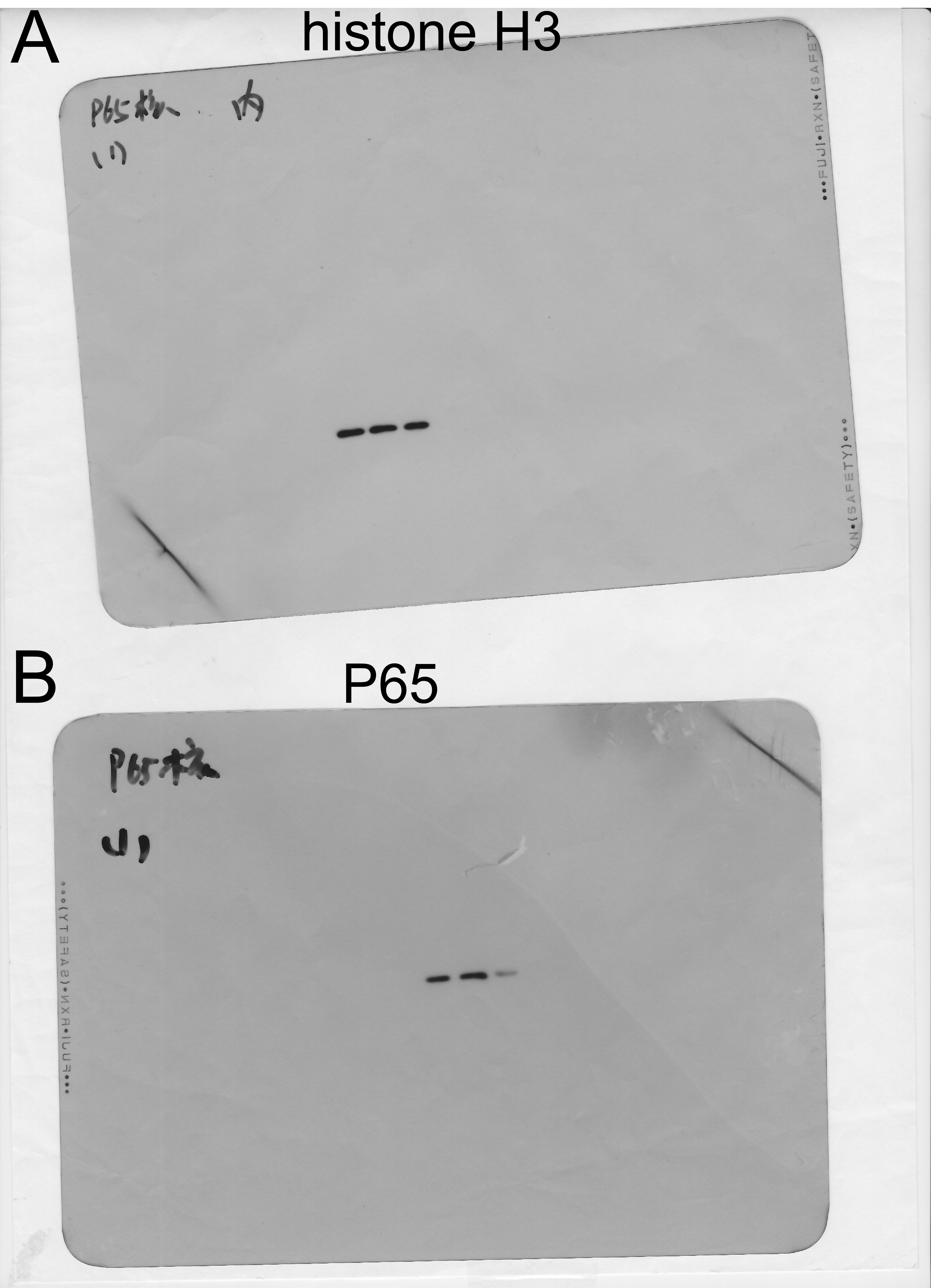

Supplement: Supplementary file 9 — Additional file 9: Figure S6. Western blot bands of histone H3 (A) and NF-κB (P65) (B) in the nucleus after hsa_circ_0043278 overexpression in MDA-MB-231 cells. [file 12885_2021_8989_MOESM9_ESM.jpg]

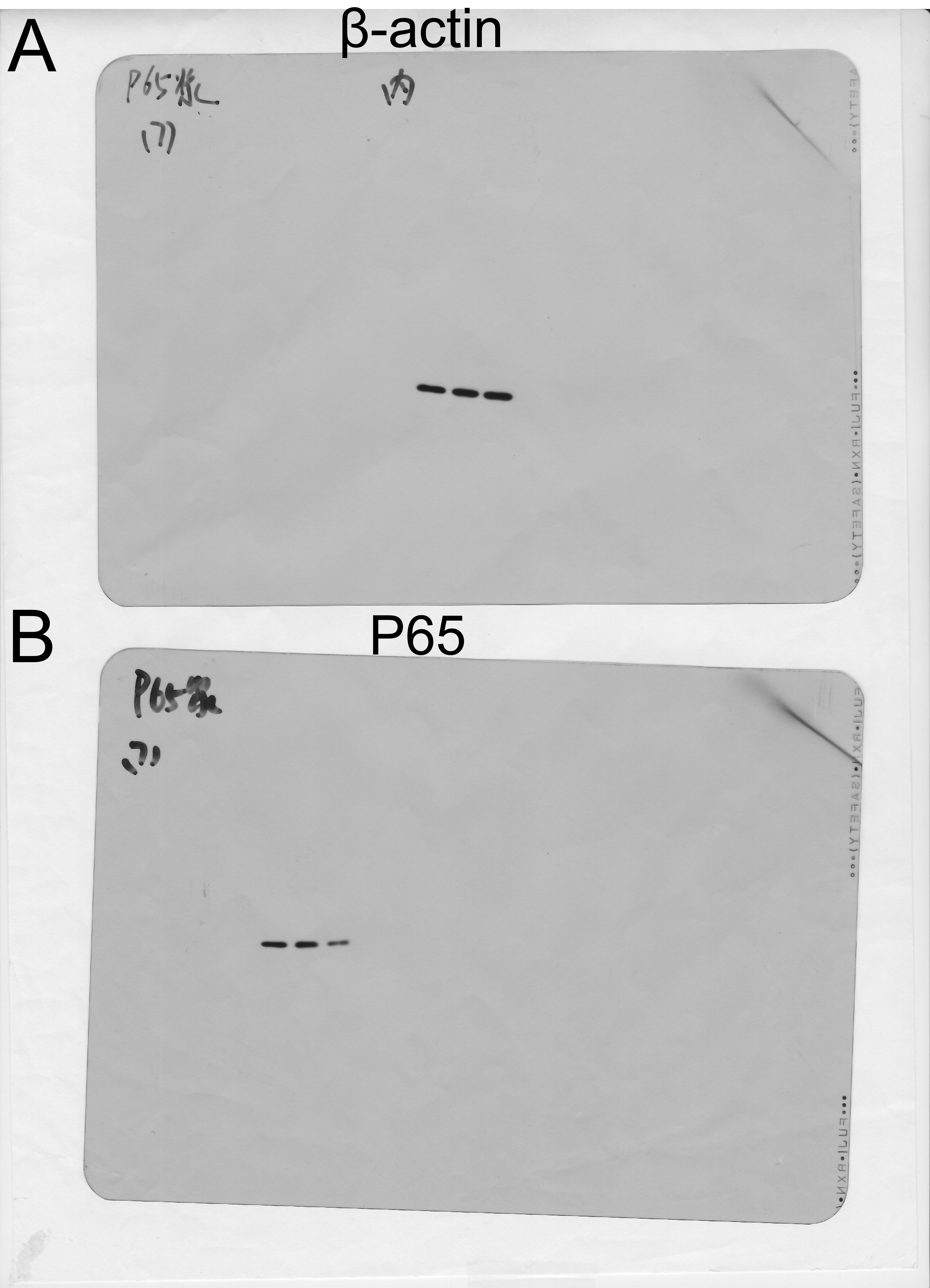

Supplement: Supplementary file 10 — Additional file 10: Figure S7. Western blot bands of β-actin (A) and NF-κB (P65) (B) in the cytosol after silencing hsa_circ_0043278 in MCF-7 cells. [file 12885_2021_8989_MOESM10_ESM.jpg]

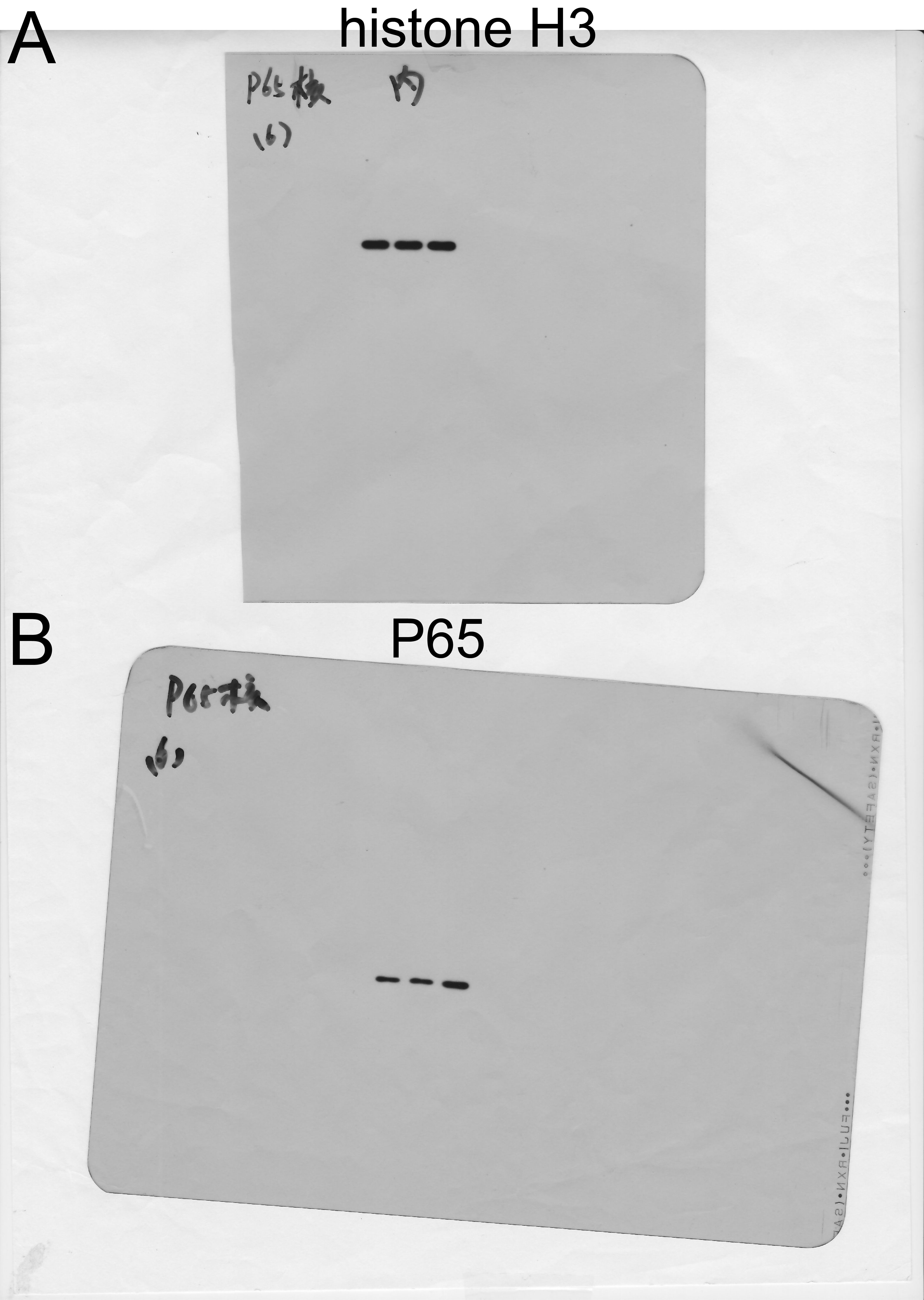

Supplement: Supplementary file 11 — Additional file 11: Figure S8. Western blot bands of histone H3 (A) and NF-κB (P65) (B) in the nucleus after silencing hsa_circ_0043278 in MCF-7 cells. [file 12885_2021_8989_MOESM11_ESM.jpg]

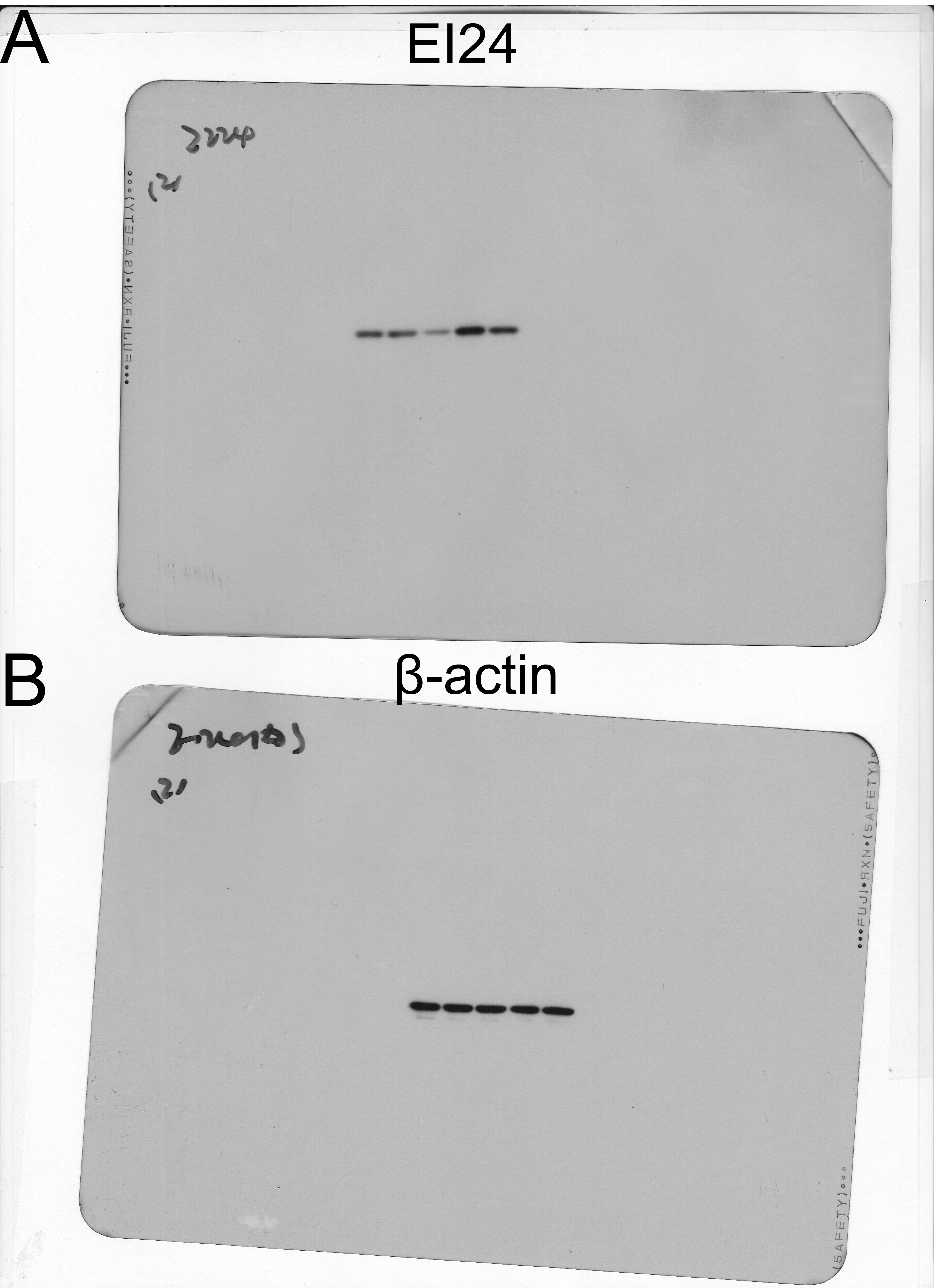

Supplement: Supplementary file 12 — Additional file 12: Figure S9. Western blot bands of EI24 (A) and β-actin (B) after hsa_circ_0043278 overexpression and/or transfection of the miR-455-3p mimic in MDA-MB-231 cells. [file 12885_2021_8989_MOESM12_ESM.jpg]

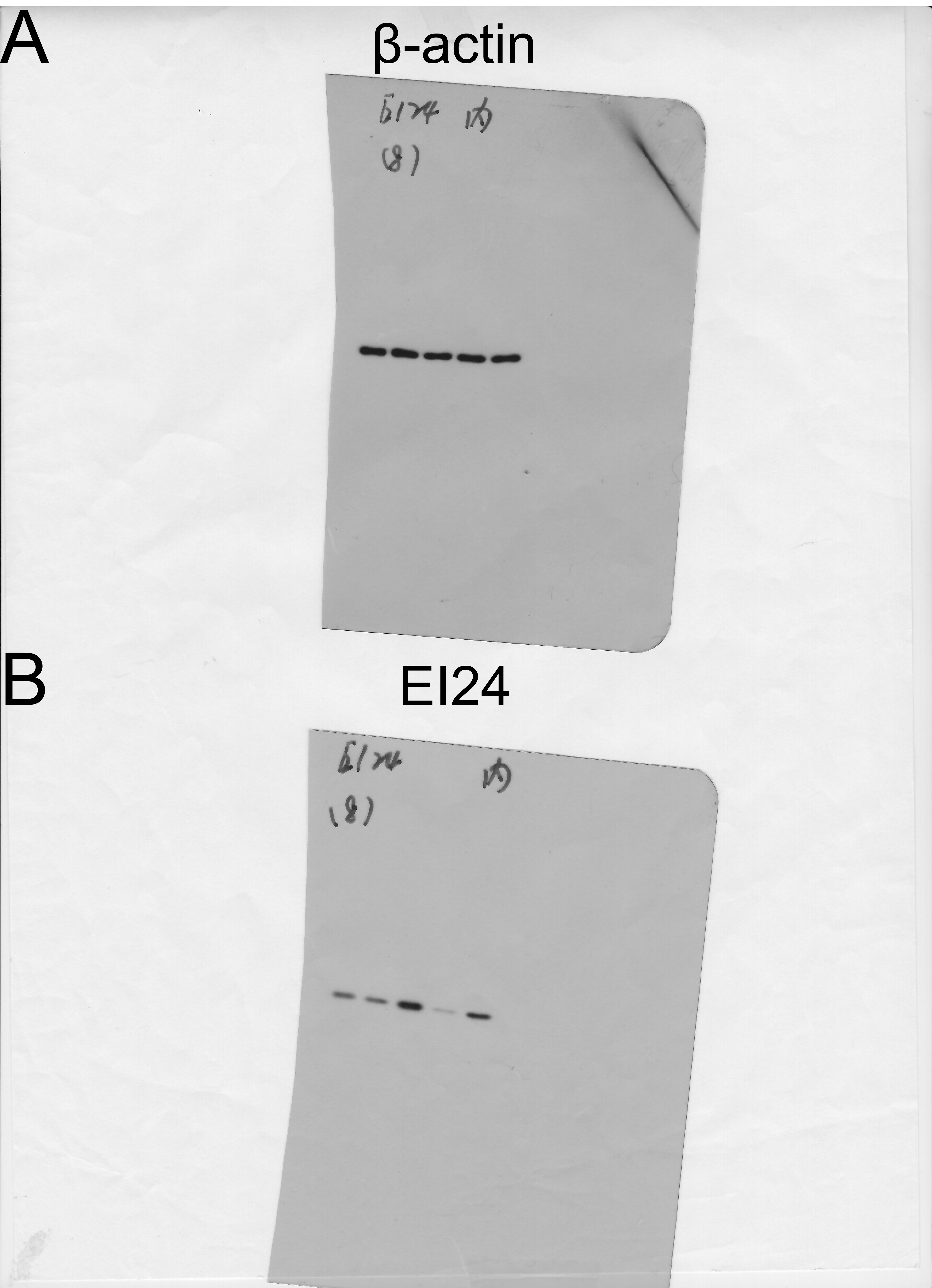

Supplement: Supplementary file 13 — Additional file 13: Figure S10. Western blot bands of β-actin (A) and EI24 (B) after hsa_circ_0043278 downregulation and/or transfection of the miR-455-3p inhibitor in MCF-7 cells. [file 12885_2021_8989_MOESM13_ESM.jpg]

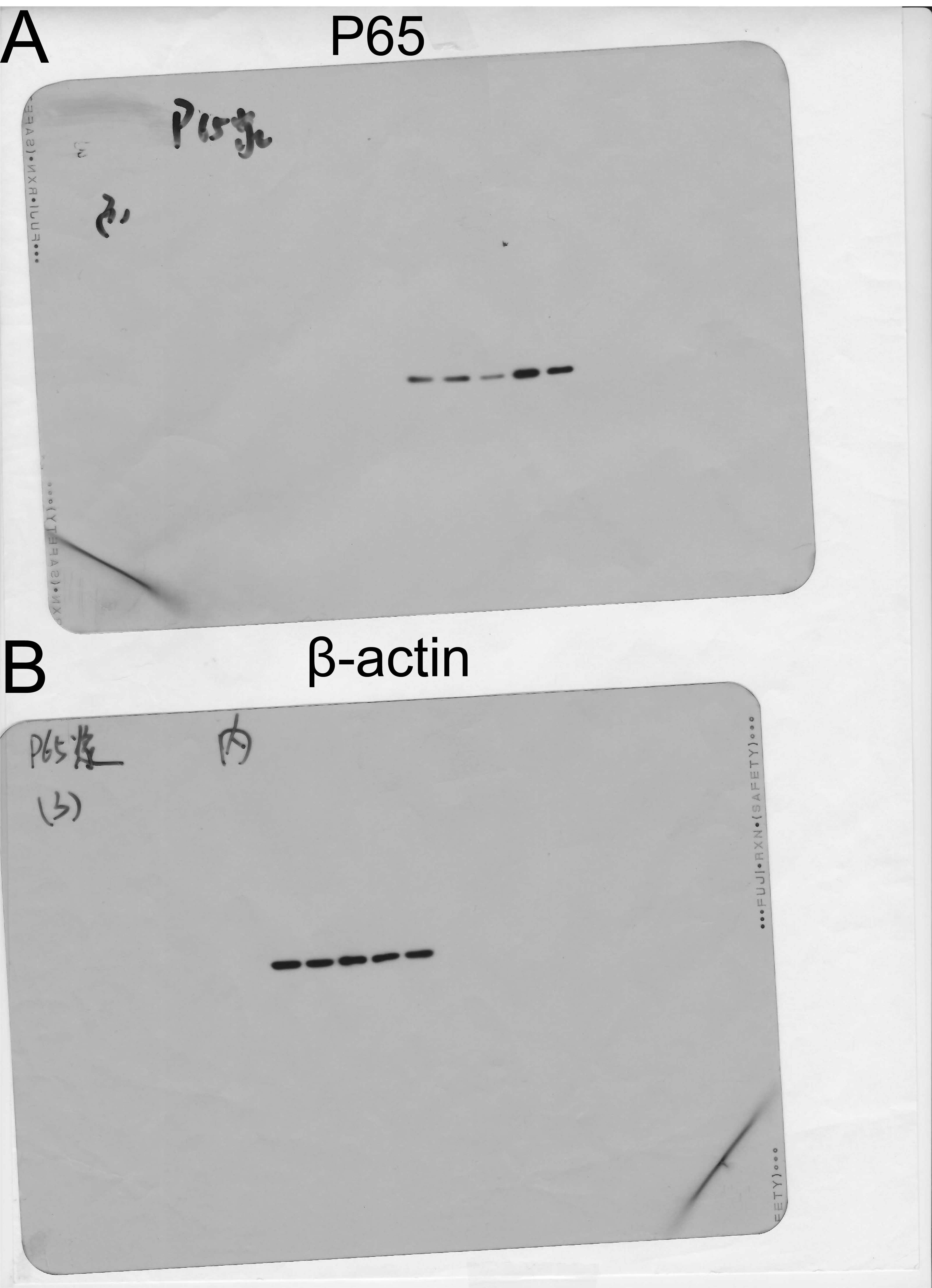

Supplement: Supplementary file 14 — Additional file 14: Figure S11. Western blot bands of NF-κB (P65) (A) and β-actin (B) in the cytosol after hsa_circ_0043278 overexpression and/or transfection of the miR-455-3p mimic in MDA-MB-231 cells. [file 12885_2021_8989_MOESM14_ESM.jpg]

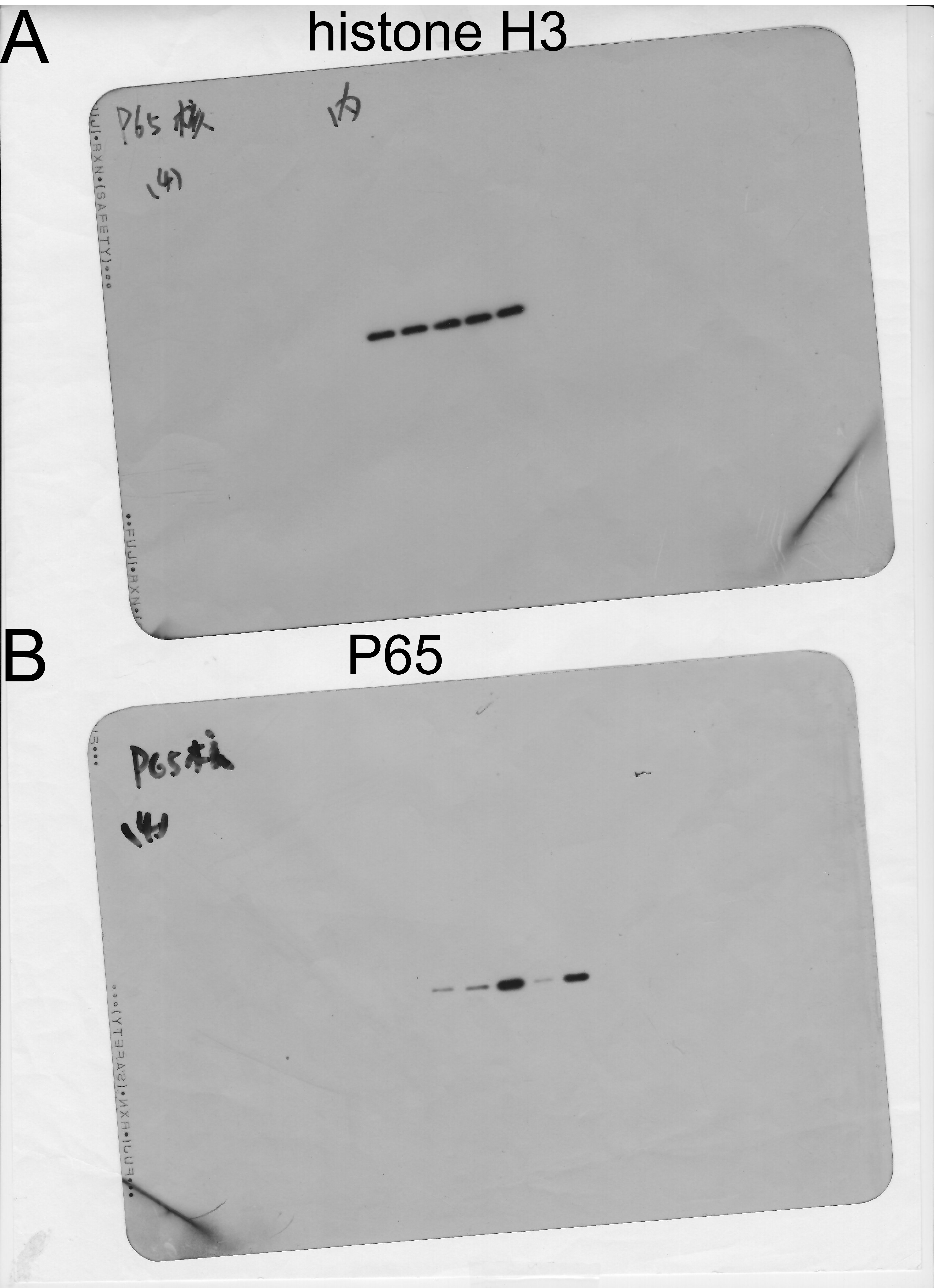

Supplement: Supplementary file 15 — Additional file 15: Figure S12. Western blot bands of histone H3 (A) and NF-κB (P65) (B) in the nucleus after hsa_circ_0043278 overexpression and/or transfection of the miR-455-3p mimic in MDA-MB-231 cells. [file 12885_2021_8989_MOESM15_ESM.jpg]

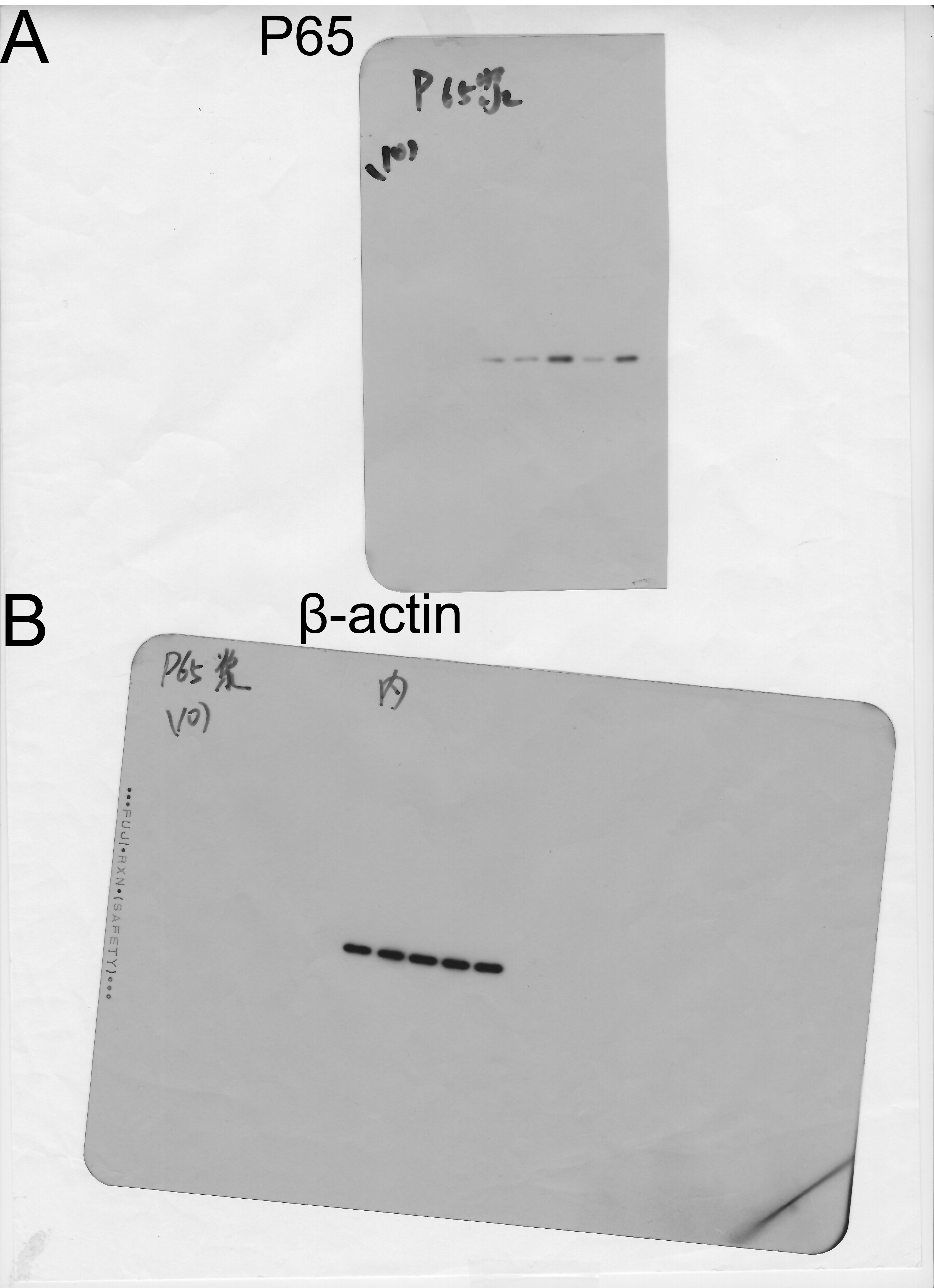

Supplement: Supplementary file 16 — Additional file 16: Figure S13. Western blot bands of NF-κB (P65) (A) and β-actin (B) in the cytosol after hsa_circ_0043278 downregulation and/or transfection of the miR-455-3p inhibitor in MCF-7 cells. [file 12885_2021_8989_MOESM16_ESM.jpg]

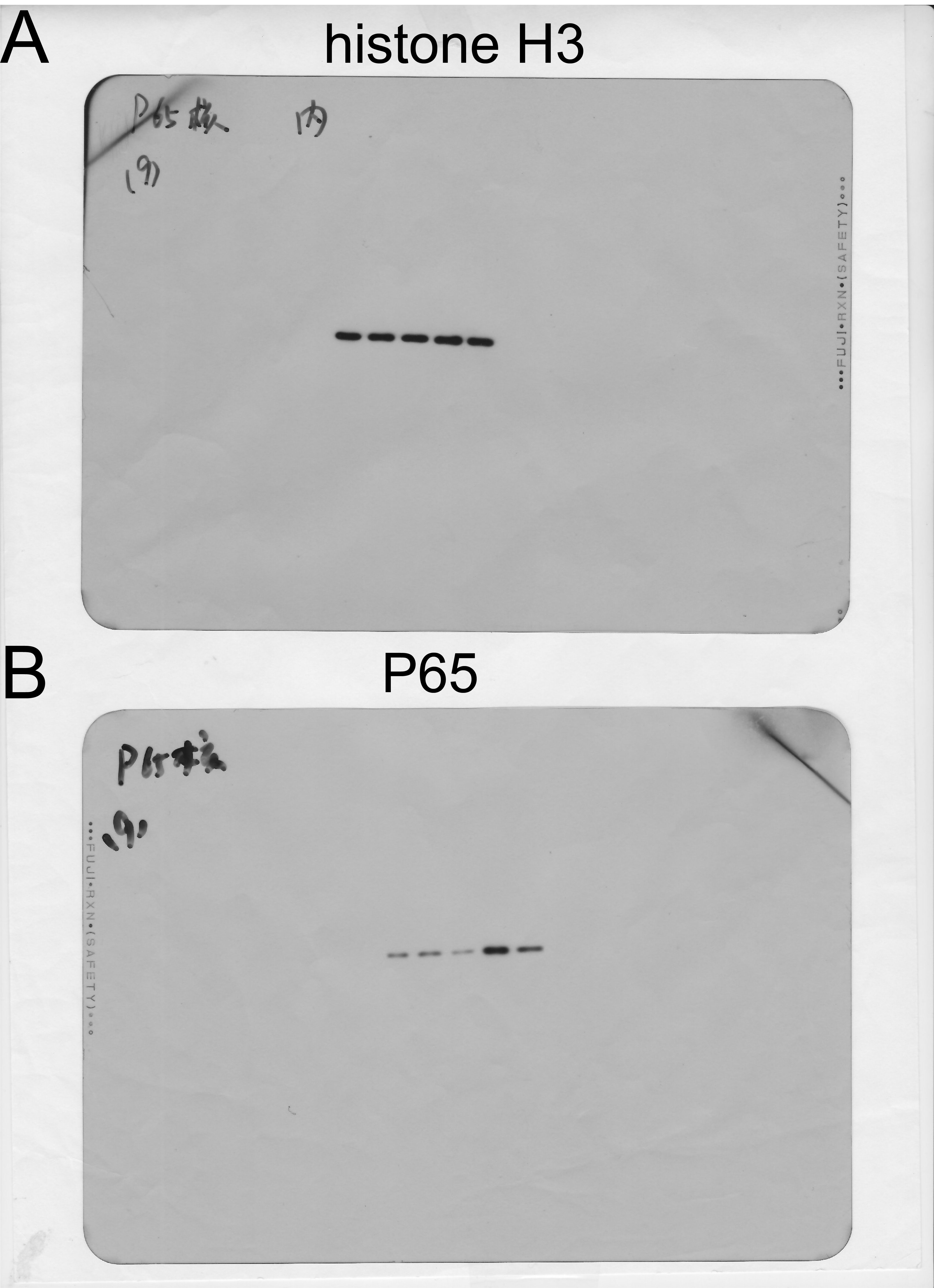

Supplement: Supplementary file 17 — Additional file 17: Figure S14. Western blot bands of histone H3 (A) and NF-κB (P65) (B) in the nucleus after hsa_circ_0043278 downregulation and/or transfection of the miR-455-3p inhibitor in MCF-7 cells. [file 12885_2021_8989_MOESM17_ESM.jpg]
